# Supplementary material for: A one-two punch targeting reactive oxygen species and fibril for rescuing Alzheimer’s disease
Source: Nat Commun. 2024 Jan 24;15:705. doi: 10.1038/s41467-024-44737-x (PMC10808243; doi:10.1038/s41467-024-44737-x)
Supplement: Supplementary file 1 — Supplementary information [file 41467_2024_44737_MOESM1_ESM.pdf]

Supplementary Information

A one-two punch targeting reactive oxygen species and fibril for rescuing Alzheimer's disease

Jiefei Wang[1,7], Ping Shangguan[1,7], Xiaoyu Chen[2], Yong Zhong[3], Ming Lin[1], Mu He[1], Yisheng Liu[1], Yuan Zhou[1], Xiaobin Pang[1], Lulu Han[1], Mengya Lu[1], Xiao Wang[1], Yang Liu[1], Huiqing Yang[1], Jingyun Chen[1], Chenhui Song[1], Jing Zhang[4]*, Xin Wang[1]*, Bingyang Shi[1,5]* & Ben Zhong Tang[6]*

[1]Henan-Macquarie Uni Joint Centre for Biomedical Innovation, Academy for Advanced Interdisciplinary Studies, Henan Key Laboratory of Brain Targeted Bio-nanomedicine, School of Life Sciences, Henan University, Kaifeng, Henan 475004, China.

[2]School of Medical Technology, Beijing Institute of Technology, Beijing, China.

[3]Key Laboratory for Special Functional Materials of Ministry of Education, National & Local Joint Engineering Research Center for High-efficiency Display and Lighting Technology, School of Materials Science and Engineering, Collaborative Innovation Center of Nano Functional Materials and Applications, Henan University, Kaifeng 475004, China.

[4]Department of Laboratory Medicine Nanfang Hospital, Southern Medical University, Guangzhou, 510515, China.

[5]Macquarie Medical School, Faculty of Medicine & Health Sciences, Macquarie University, Sydney, NSW 2109, Australia.

[6]School of Science and Engineering, Shenzhen Institute of Aggregate Science and Technology, The Chinese University of Hong Kong, Shenzhen, Guangdong 518172, China.

[7]These authors contributed equally.

Email: zhangjingzisefeng@163.com (J. Zhang); wx@henu.edu.cn (X. Wang); bingyang.shi@mq.edu.au (B. Shi); tangbenz@cuhk.edu.cn (B. Z. Tang).

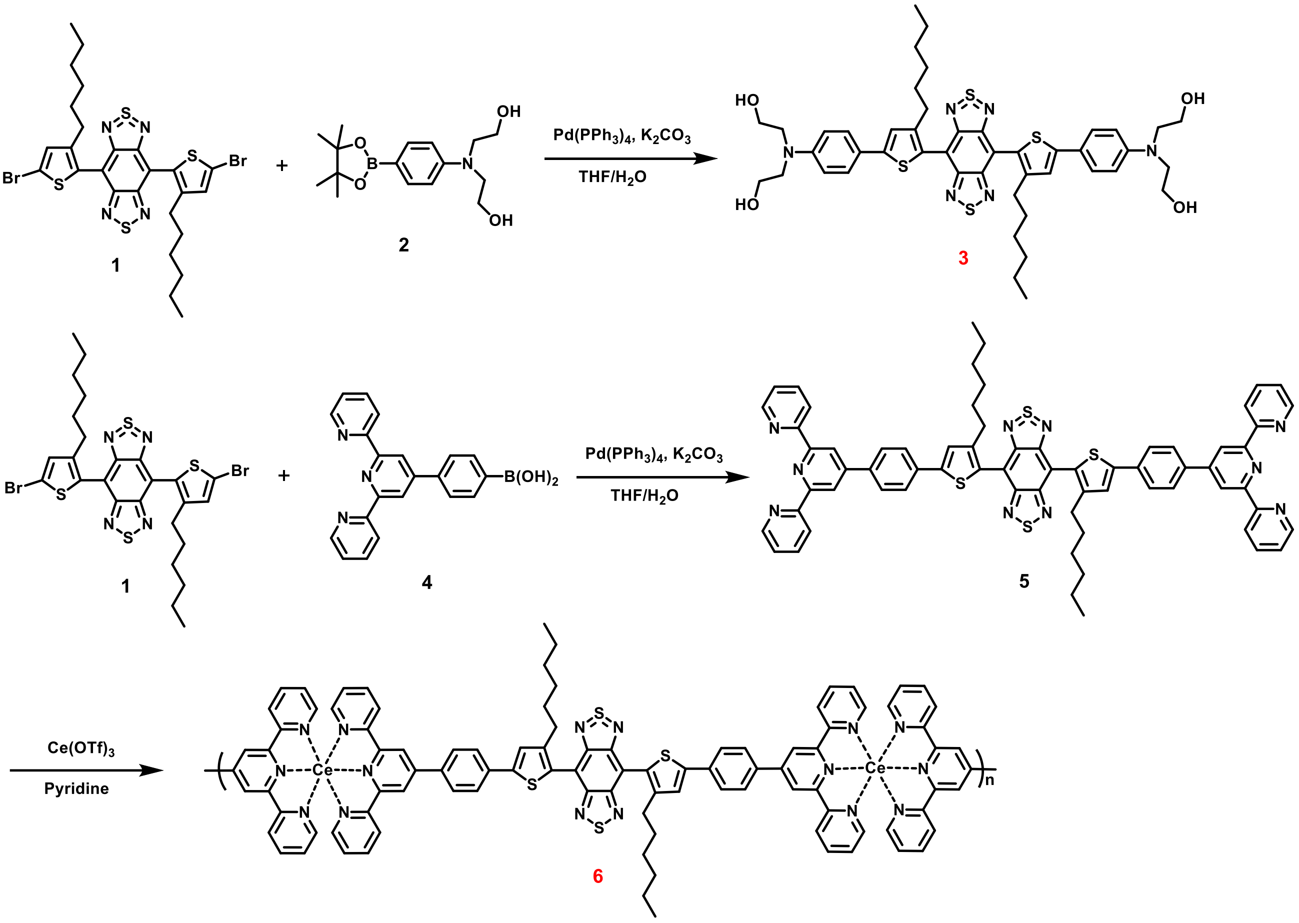

Supplementary Fig. 1 Synthetic route of the target compounds **3** and **6**.

Syntheses of the intermediate and the target compounds

All the starting materials were purchased from Henan Lien Chemical Products Co., Ltd. All reagents were analytical grade and used as received without further purification. All manipulations were carried out under a dry argon gas atmosphere by using standard Schlenk techniques, unless stated otherwise. Solvents were pre-dried and distilled under argon prior to use, except those used directly for spectroscopic measurements, which were of spectroscopic grade.

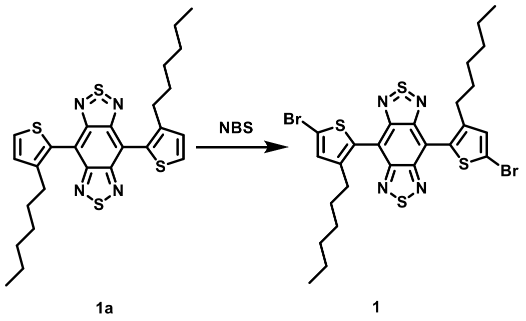

Supplementary Fig. 2 Synthetic route of compound **1**.

Synthesis of **1**: Compound **1** was prepared according to the procedures described in the corresponding literature (J. Am. Chem. Soc. 2019, 141, 13, 5359–5368). The starting material **1a** was purchased from Henan Lien Chemical Products Co., Ltd. Under N_2 atmosphere, **1a** (0.3 g, 0.33 mmol) was dissolved in mixture of 10 mL $CHCl_3$ and 10 mL acetic acid. Then, under dark conditions at room temperature, slowly add a mixture of 5 mL $CHCl_3$ and 5 mL acetic acid containing *N*-bromosuccinimide (NBS) (117 mg, 6.6 mmol) to the above mixture. The mixture is stirred overnight and then dried with condensed air. Then the crude product was purified by silica gel column to obtain the target molecule (yield, 45%). ^{1}H NMR (400 MHz, $CDCl_3$) δ 7.21 (s, 2H), 2.52 (t, J = 7.6 Hz 4H), 1.62-1.54 (m, 4H), 1.17-1.03 (m, 12H), 0.75 (t, J = 6.8 Hz, 6H). ^{13}C NMR (100 MHz, $CDCl_3$) δ 153.0, 145.1, 132.0, 130.3, 115.5, 115.4, 31.4, 30.2, 23.0, 28.9, 22.4, 13.9.

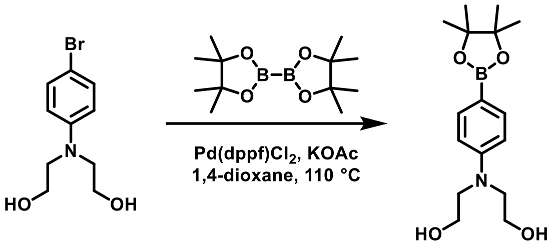

Supplementary Fig. 3 Synthetic route of compound **2**.

Synthesis of **2**: Compound **2** was prepared according to the procedures described in the corresponding literature (J. Med. Chem. 2014, 57, 11, 4498-4510). The starting material **2a** was purchased from Henan Lien Chemical Products Co., Ltd. Under N_2 atmosphere, **2a** (0.52 g, 2 mmol), bis(pinacolato)diboron (0.762 g, 3 mmol), KOAc (0.588 g, 6 mol), and Pd(dppf)Cl_2 (0.146 g, 0.2 mmol) was dissolved in dioxane (20 mL) and heated to reflux overnight. After cooling to room temperature, the mixture was extracted with ethyl acetate and washed with brine. The organic layers were collected and dried over Na_2SO_4. After evaporation of the solvent, the product was dried in vacuum and used directly without further purification.

Synthesis of 3

To a solution of compound **1** (1.00 mmol) and compound **2** (2.20 mmol) in THF/water (v/v: 5:1) mixture (60 mL) was added $Pd(PPh_3)_4$ (0.10 mmol) and K_2CO_3 (3.00 mmol). The mixture was stirred at 70 °C under N_2 for 24 h. Then the reaction was quenched by the addition of water (150 mL) and extracted with CH_2Cl_2 (3 × 50 mL). The combined organic extract was dried over anhydrous Na_2SO_4 and filtered. The solvent was removed by rotary evaporation. The product was purified by column

chromatography (silica gel, ethyl acetate) to obtain compound **3** as a green solid. Yield: 18%.^{1}H NMR (400 MHz, $CDCl_3$), *δ* (ppm): 7.57-7.46 (m, 4H), 7.29 (s, 1H), 7.15 (s, 1H), 6.70-6.65 (m, 4H), 4.22 (m, 4H), 3.84 (d, *J* = 4 Hz, 8 H), 3.59 (d, *J* = 4 Hz, 8 H), 2.56 (t, *J* = 8 Hz, 2H), 2.40-2.34 (m, 2H), 1.64-1.52 (m, 4H), 1.12-1.07 (m, 12H), 0.77-0.70 (m, 6H). ^{13}C NMR (100 MHz, $CDCl_3$), *δ* (ppm): 153.2, 151.4, 147.6, 147.3, 145.6, 145.4, 144.0, 143.7, 140.3, 127.1, 126.9, 125.9, 125.7, 123.7, 123.3, 123.1, 123.0, 116.0, 112.6, 112.5, 106.2, 60.7, 55.2, 31.5 (m), 30.5, 30.2, 29.1 (m), 22.4, 14.0. HRMS (MALDI-TOF), *m*/*z*: $[M+H]^+$ calcd. for $C_{46}H_{57}N_6O_4S_4$: 885.3324; found: 885.3326.

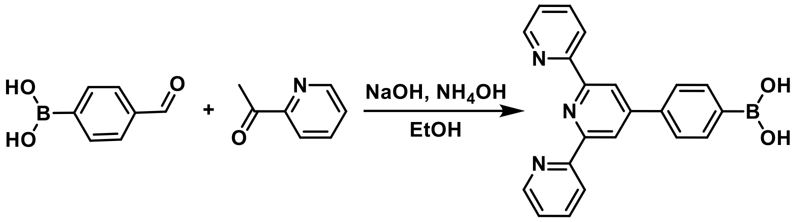

4

Supplementary Fig. 4 Synthetic route of compound **4**.

Synthesis of **4**: Compound **4** was prepared according to the procedures described in the corresponding literature (ACS Macro Lett. 2020, 9, 11, 1655–1661). The starting materials 4-formylphenylboronic acid and 2-acetylpyridine were purchased from Henan Lien Chemical Products Co., Ltd. A mixture of 4-formylphenylboronic acid (1 g, 6.7 mmol), 2-acetylpyridine (2.7 g, 21.9 mmol), NaOH (1.1 g, 26.3 mmol) and EtOH (50 mL) were added. After stirring for 8 h at room temperature. Then, the mixture was heated to 65 ºC for 12 h after 25 mL concentrated ammonia solution added. During the reaction, a large amount of precipitate was produced. After cooling to room temperature, the product was collected by filtering, washed with lots of isopropanol and $CHCl_3$, and dried in vacuum. Then the product was used directly without further purification.

Synthesis of compound 5

A similar procedure to the synthesis of compound **3** was employed. The crude product was recrystallized from CH_2Cl_2/diethyl ether to give a green solid in 62% yield. ^{1}H NMR (400 MHz, $CDCl_3$), *δ* (ppm): 8.79-8.67 (m, 9H), 7.98-7.86 (m, 6H), 7.79 (d, *J* = 8 Hz, 2H), 7.66 (d, *J* = 8 Hz, 2H), 7.47 (d, *J* = 4 Hz, 1H), 7.39-7.34 (m, 6H), 7.22 (t, *J* = 8 Hz, 2H), 7.14-7.11 (m, 3H), 4.27 (m, 2H), 2.50-2.46 (m, 2H), 1.65 (m, 4H), 1.25-1.19 (m, 12H), 0.88-0.78 (m, 6H). ^{13}C NMR (100 MHz, $CDCl_3$), *δ* (ppm): 156.2, 156.0, 149.5, 149.1, 144.5, 144.2, 140.1, 137.4, 136.9, 136.0, 135.1, 134.1, 130.9, 128.3, 127.8, 126.1, 123.8, 121.4, 118.5, 31.6 (m), 30.3 (m), 29.3 (m), 29.0, 22.5, 14.0. HRMS (MALDI-TOF), *m*/*z*: $[M+H]^+$ calcd. for $C_{68}H_{57}N_{10}S_4$: 1141.3650; found: 1141.3654.

Synthesis of 6

A mixture of Compound **5** (1.00 mmol) and Ce(OTf$_3$) (1.00 mmol) was stirred in ultra-dry pyridine (20 mL) for 24 h under N_2 at room temperature. After the reaction was completed, the mixture was concentrated to 2 mL, and further poured into the mixed solvent of THF/diethyl ether (20 mL, v/v = 1:1). The precipitate was collected and dried to obtain light green solid target product compound **6** (Yield: 42%). ^{1}H NMR (400 MHz, DMSO-d_6), δ (ppm): 8.80-8.66 (m, 11H), 8.03 (t, J = 8 Hz, 3H), 7.95-7.83 (m, 8H), 7.62-7.40 (m, 8H), 2.41-2.33 (br, 4H), 1.49 (br, 4H), 1.08-1.03 (m, 12H), 0.71-0.65 (M, 6H). ^{13}C NMR (100 MHz, DMSO-d_6), δ (ppm): 155.3, 150.9, 149.0, 142.0, 138.2, 138.0, 135.8, 135.2, 131.6, 128.9 127.6, 125.9, 124.8, 122.3, 121.3, 119.1, 117.8, 30.9 (d), 29.3 (d), 28.7, 28.1 (d), 21.9, 13.8 (d).

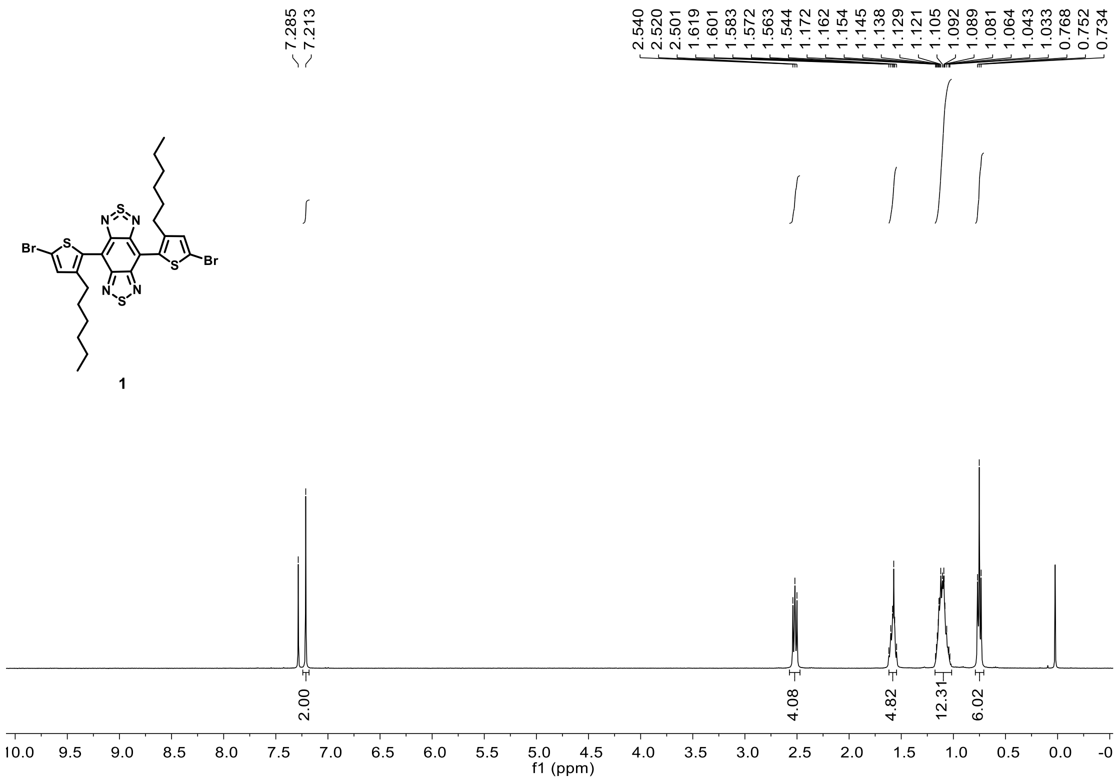

Supplementary Fig. 5 ^{1}H NMR spectrum of compound **1** in $CDCl_3$.

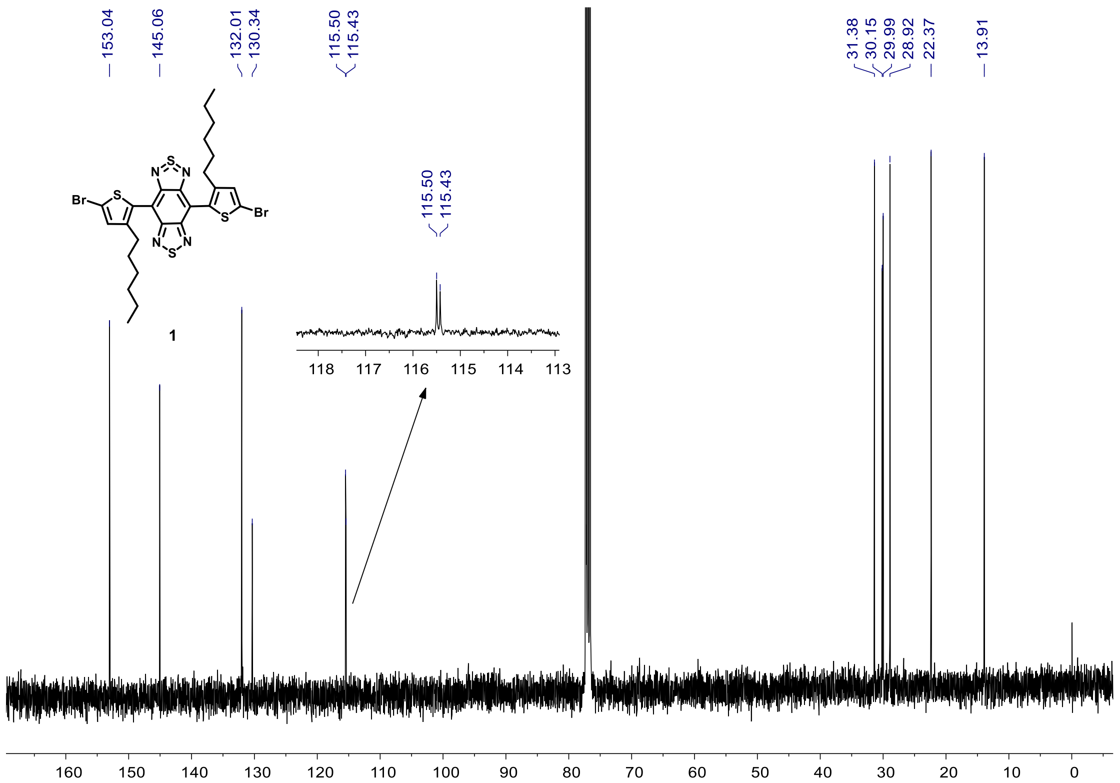

Supplementary Fig. 6 ^{13}C NMR spectrum of compound **1** in $CDCl_3$.

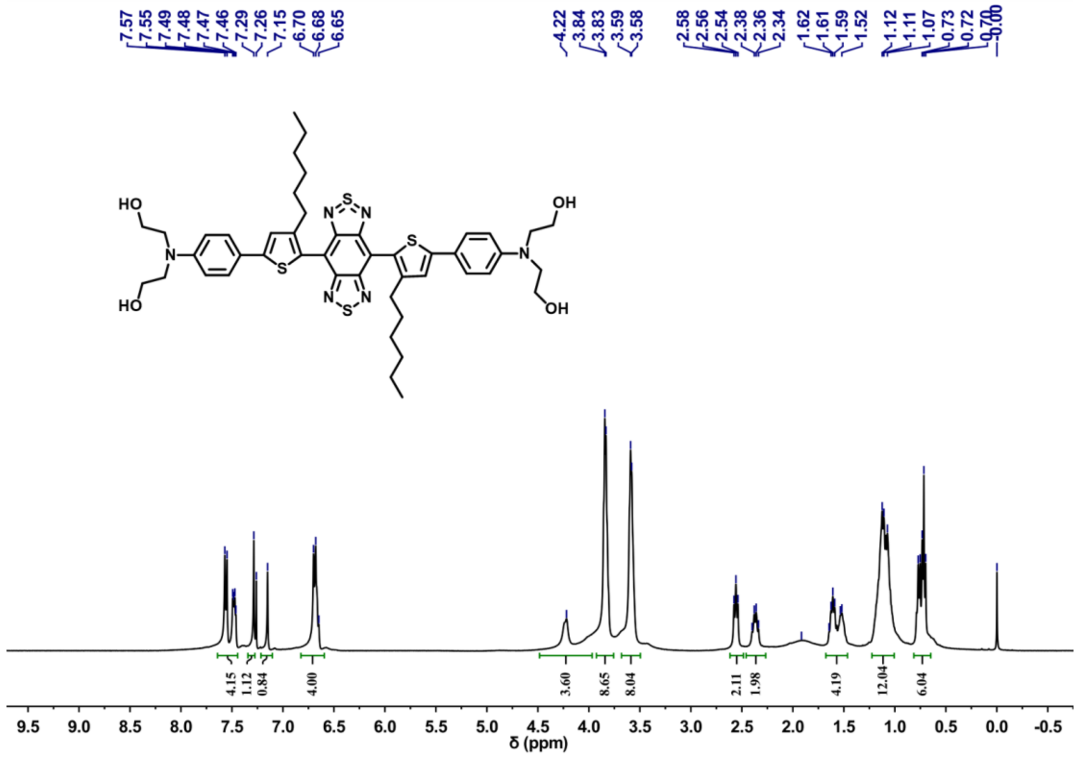

Supplementary Fig. 7 ^{1}H NMR spectrum of compound **3** in $CDCl_3$.

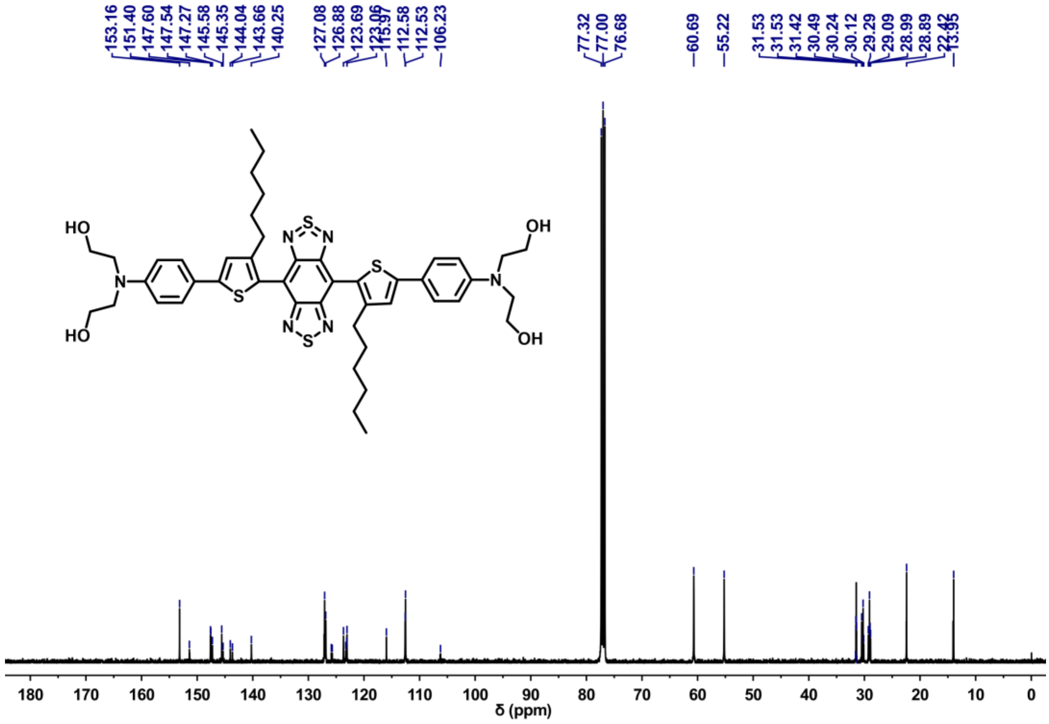

Supplementary Fig. 8 ^{13}C NMR spectrum of compound **3**.

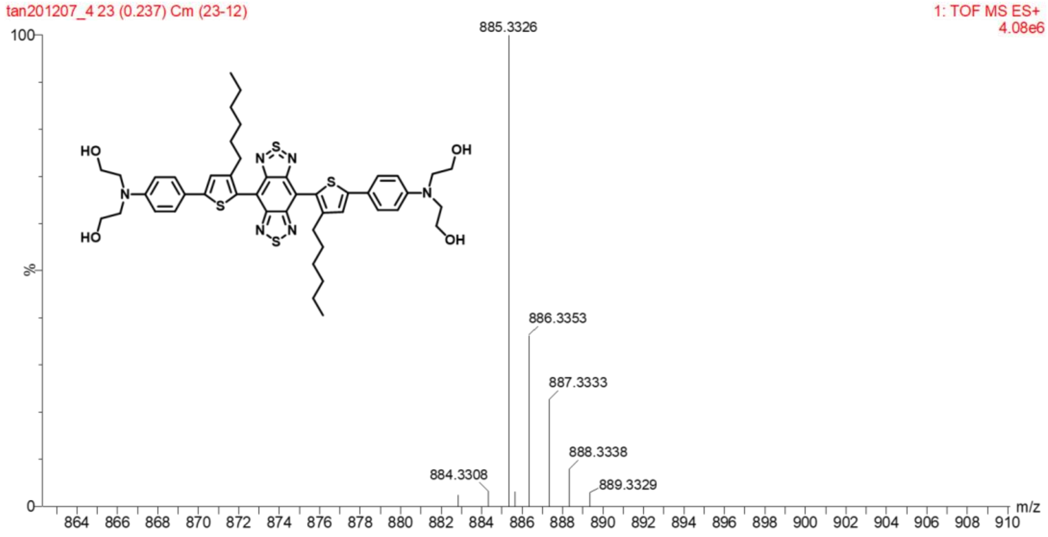

Supplementary Fig. 9 HR-MS spectrum of compound **3**.

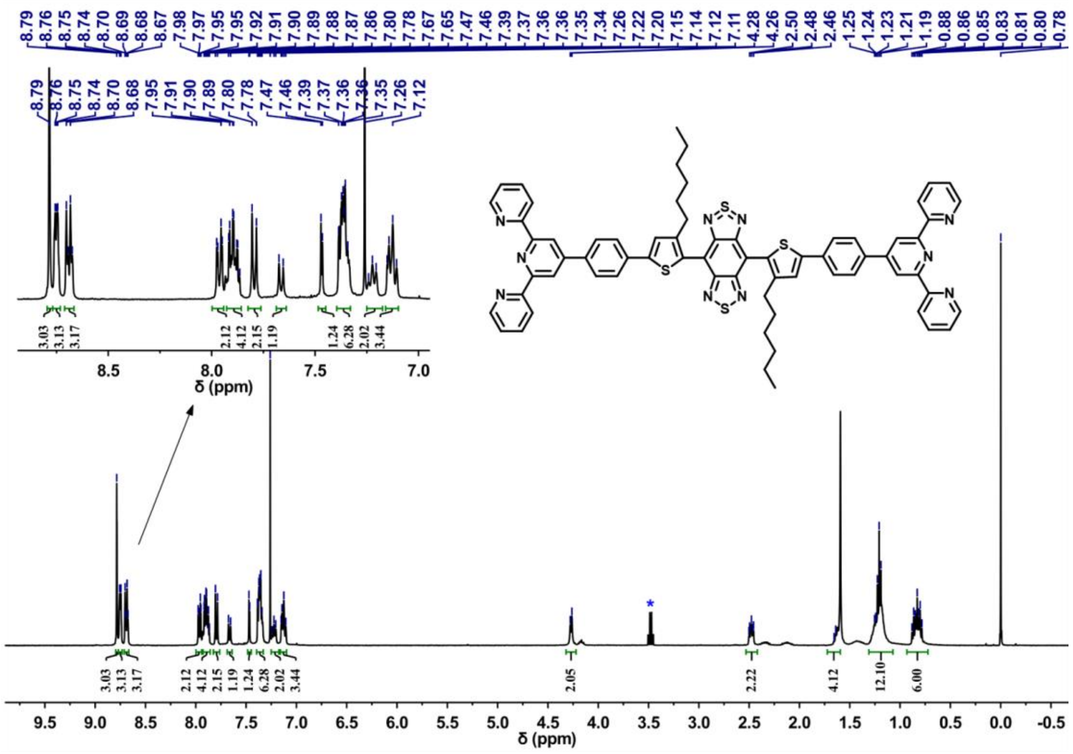

Supplementary Fig. 10 ^{1}H NMR spectrum of compound **5**.

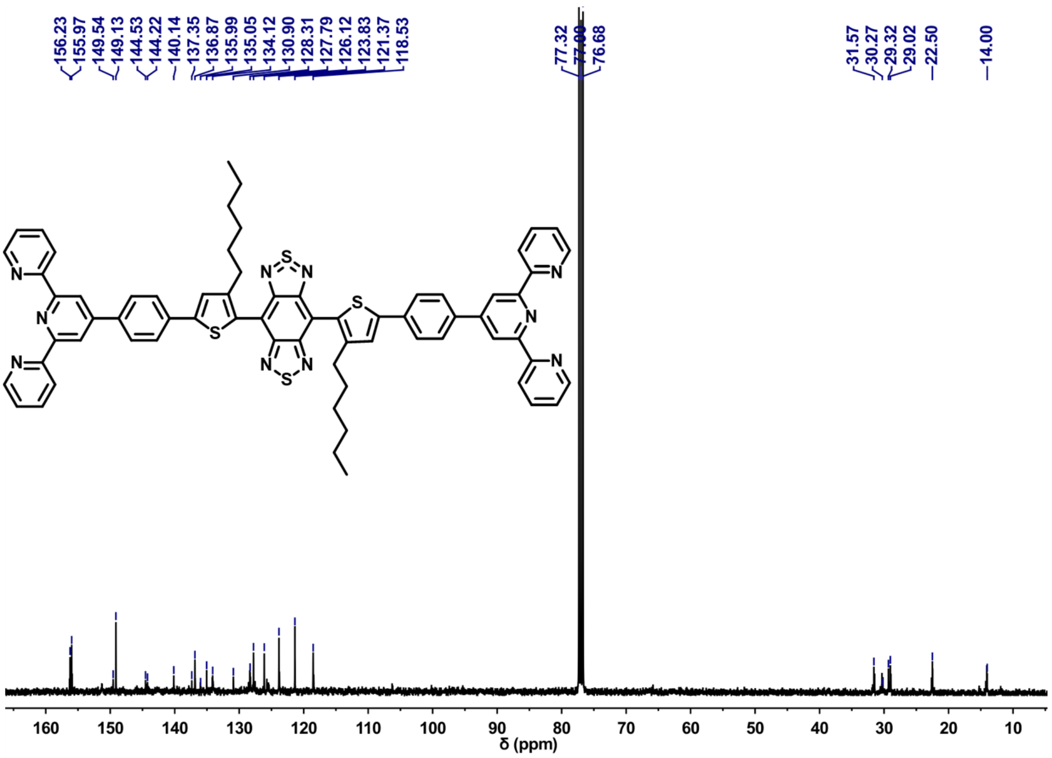

Supplementary Fig. 11 ^{13}C NMR spectrum of compound **5**.

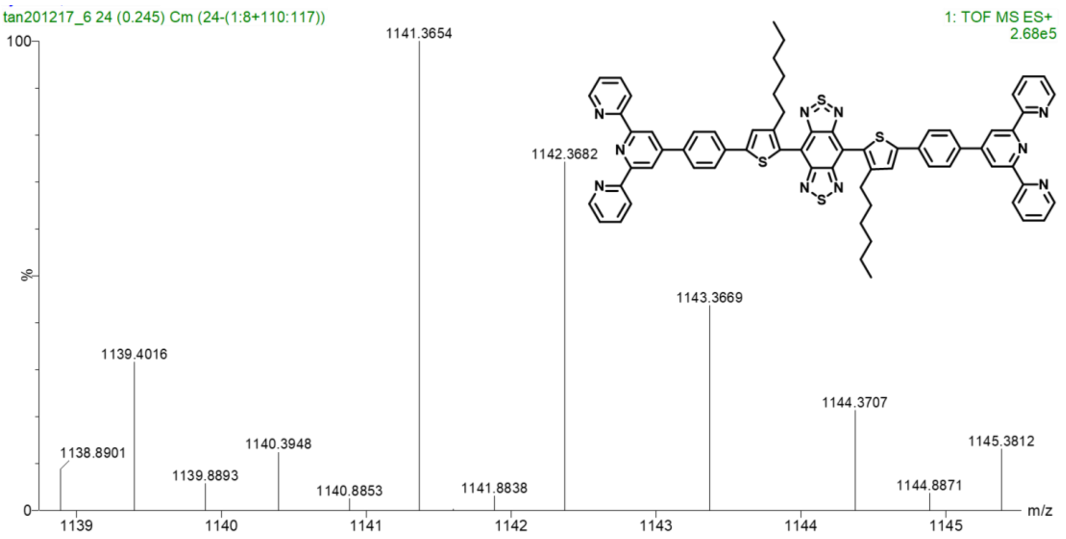

Supplementary Fig. 12 HR-MS spectrum of compound **5**.

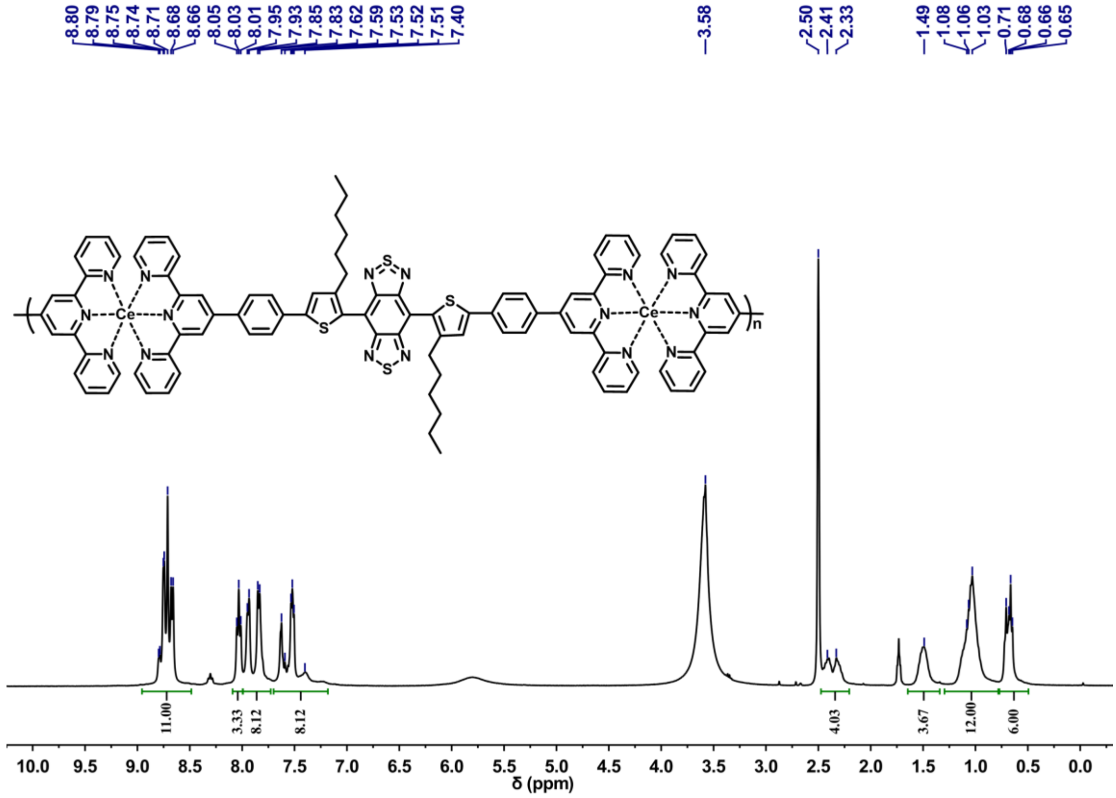

Supplementary Fig. 13 ^{1}H NMR spectrum of compound **6**.

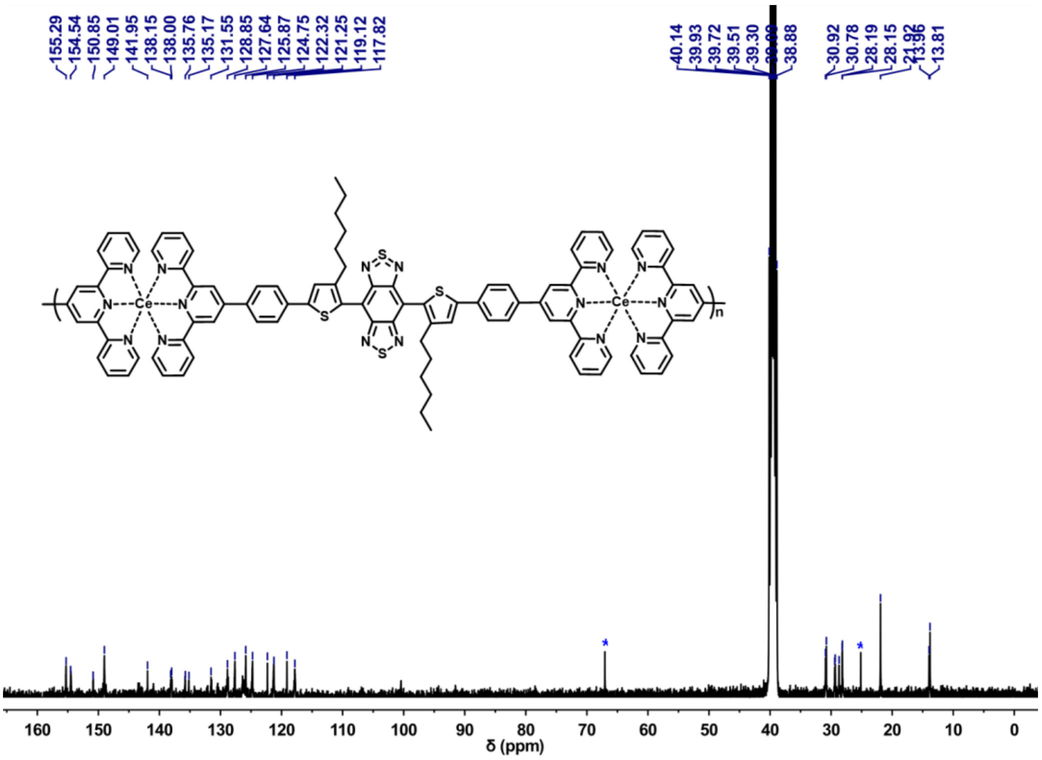

Supplementary Fig. 14 ^{13}C NMR spectrum of compound **6**.

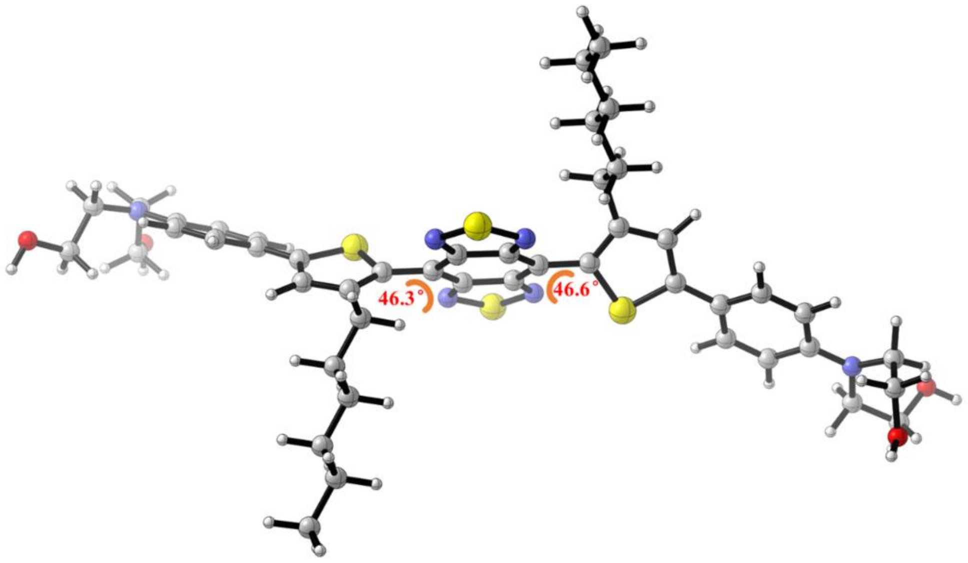

Supplementary Fig. 15 Optimized S_0 geometry detail of compound **3**.

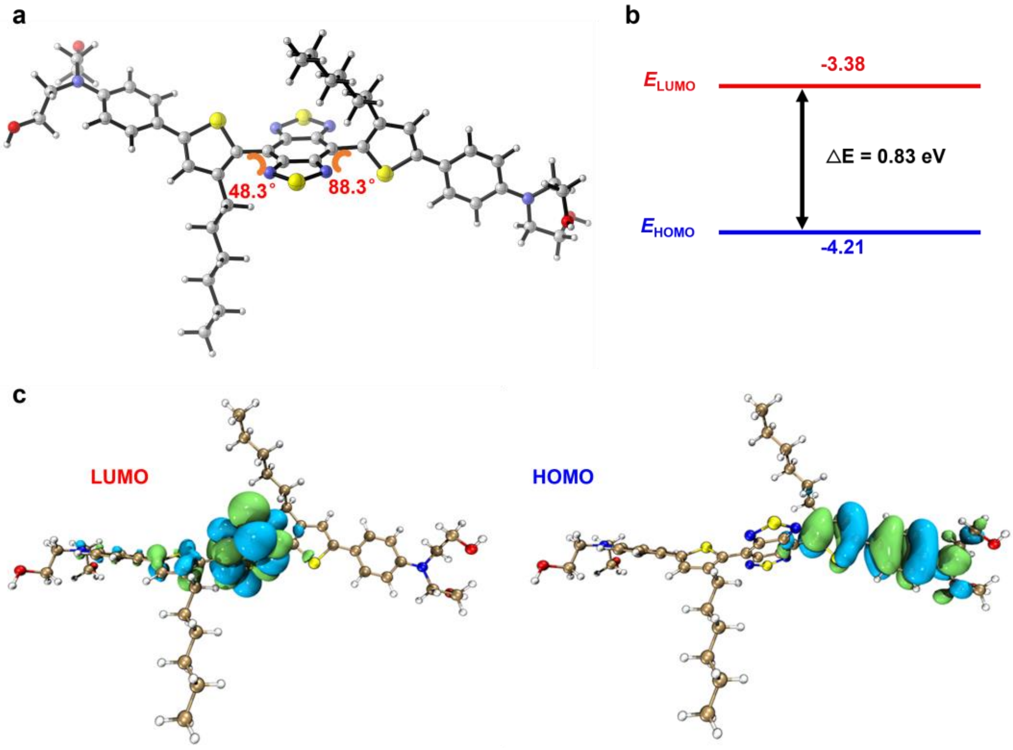

Supplementary Fig. 16 a Optimized S_1 geometry of **3** in S_1 state. **b** Energy gap between the lowest unoccupied molecular orbital (LUMO) and the highest occupied molecular orbital (HOMO) of **3**. **c** Illustration of the frontier molecular orbitals (LUMO and HOMO).

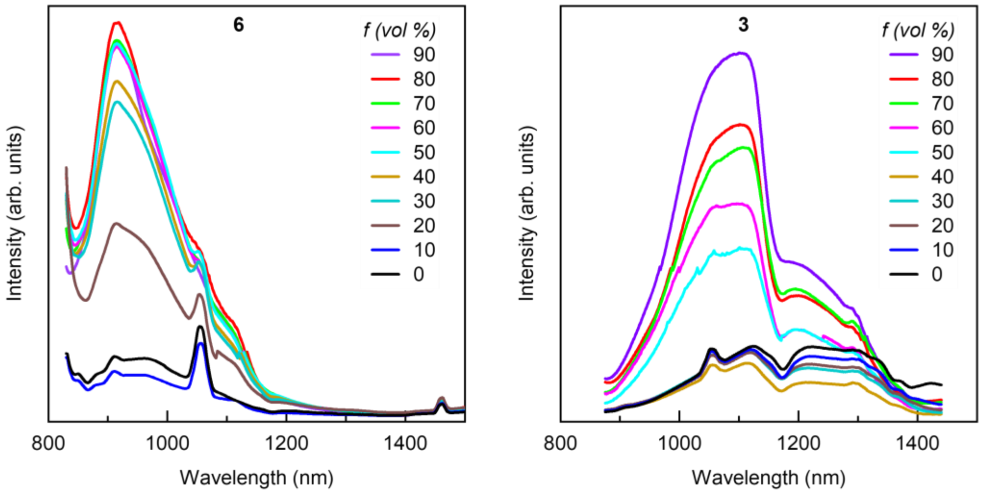

Supplementary Fig. 17 Fluorescence spectra of **6** and **3** with various fractions of deionized water.

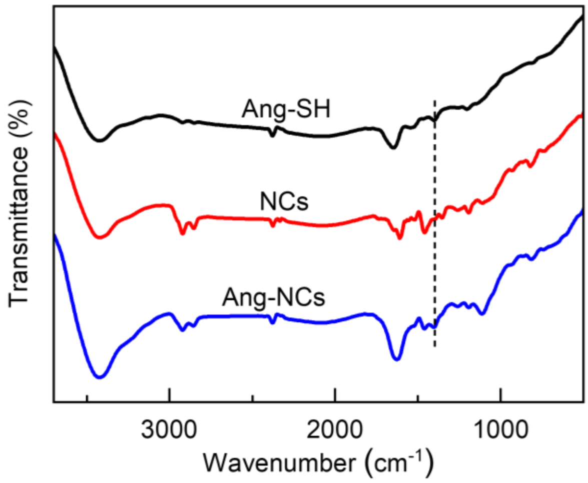

Supplementary Fig. 18 Fourier transform infrared (FTIR) spectra comparison among Ang-SH peptide, NCs, and Ang-NCs.

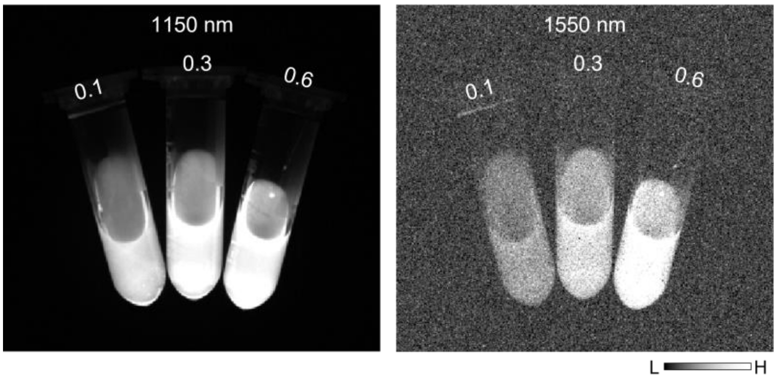

Supplementary Fig. 19 Near-infrared (NIR) images of NCs aqueous solutions under 1150 nm and 1550 nm filters.

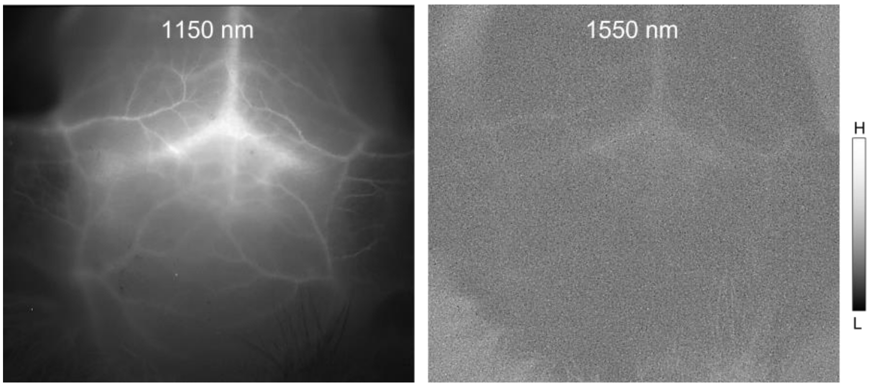

Supplementary Fig. 20 *In vivo* NIR imaging of cerebral vessel under 1150 nm and 1550 nm filters.

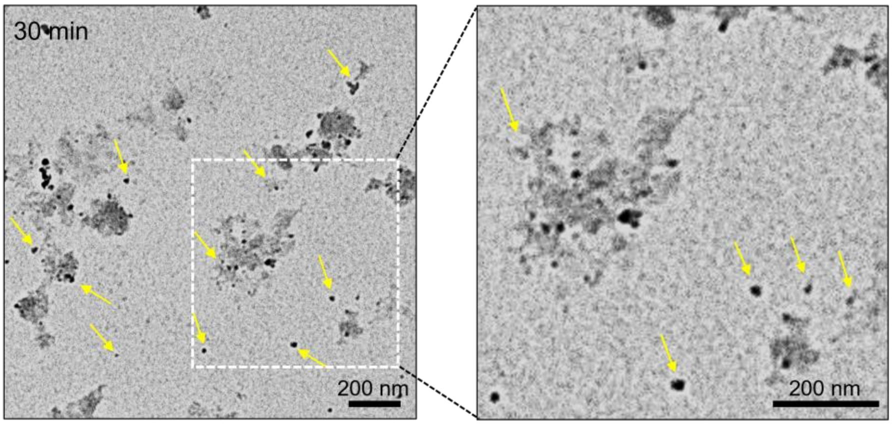

Supplementary Fig. 21 TEM image and corresponding amplifying profile of the NCs after H_2O_2 treatment for 30 min.

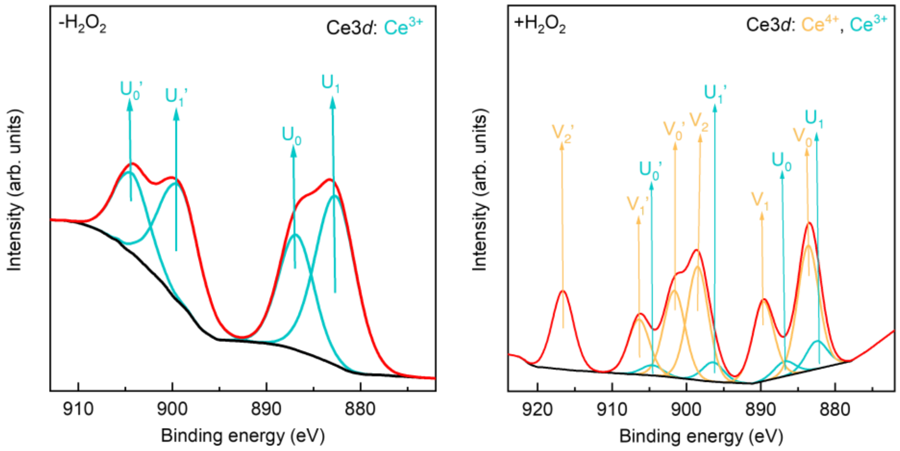

Supplementary Fig. 22 X-ray photoelectron spectroscopy (XPS) of NCs before (left) and after (right) the treatment of hydrogen peroxide (H_2O_2).

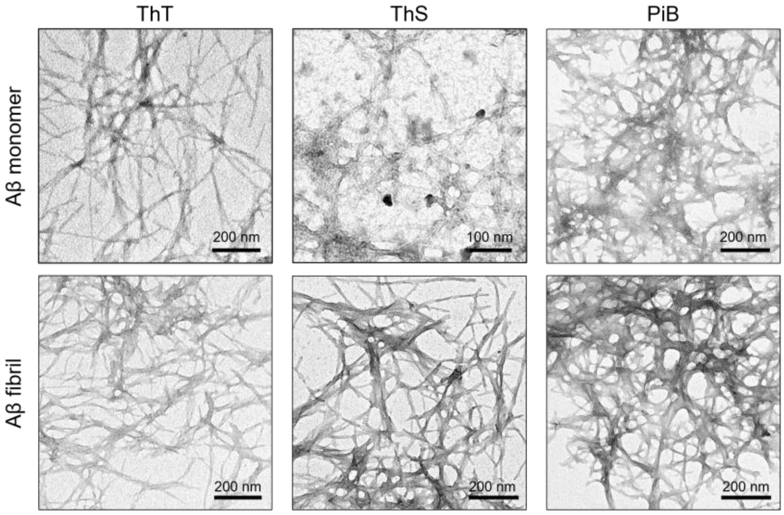

Supplementary Fig. 23 TEM images of Aβ monomer (25 μM) and Aβ fibril (25 μM) respectively incubated with ThT (25 μM), ThS (25 μM), and PiB (25 μM).

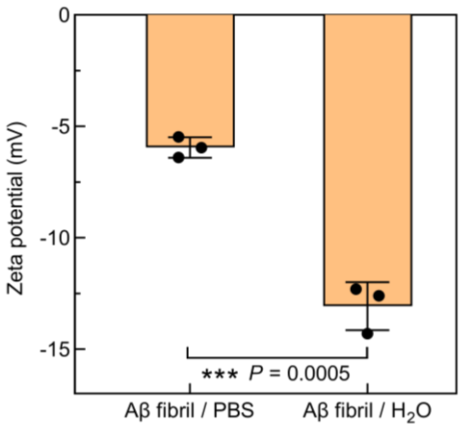

Supplementary Fig. 24 Zeta potential comparison between the Aβ fibril dispersed in PBS and that in H_2O. Data was presented as mean ± SD. ****P* < 0.001, two-tailed Student's *t*-test.

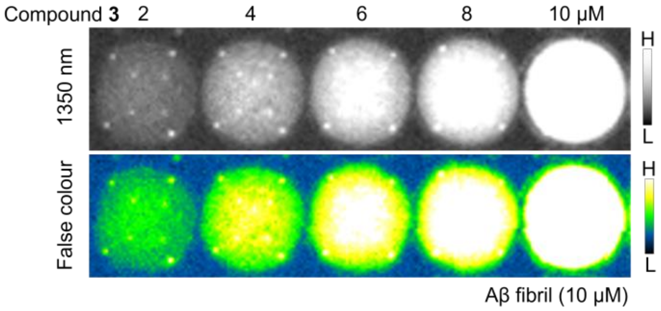

Supplementary Fig. 25 NIR image at 1350 nm and corresponding false color image of Aβ fibril (10 μM) with various concentrations of **3** (2, 4, 6, 8, and 10 μM).

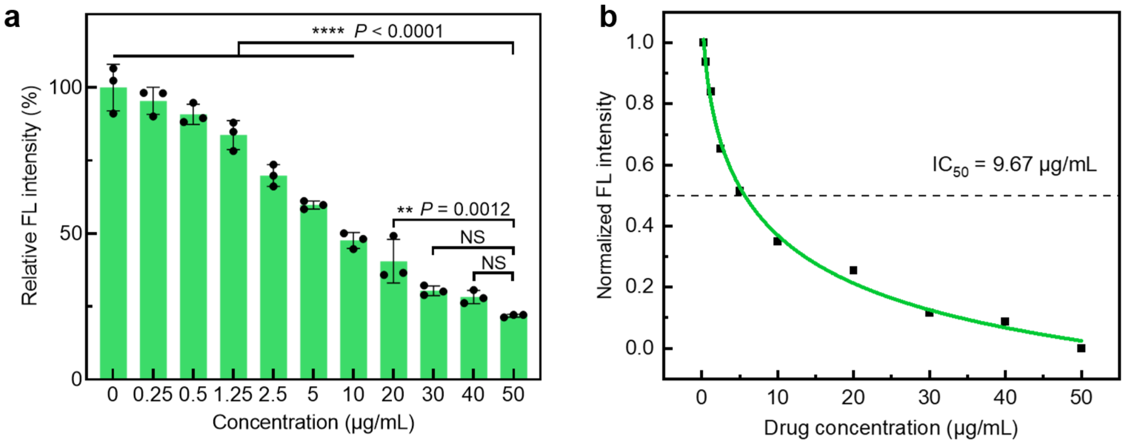

Supplementary Fig. 26 **a** Relative fluorescence intensity of ThT (5 μM) in the mixture of Aβ monomer (10 μM) with various concentrations of NCs. **b** Inhibition kinetic curve and IC_{50} value for NCs against Aβ monomer. Data was presented as mean ± SD. NS, no significance. ***P* < 0.01, *****P* < 0.0001, one-way analysis of variance (ANOVA).

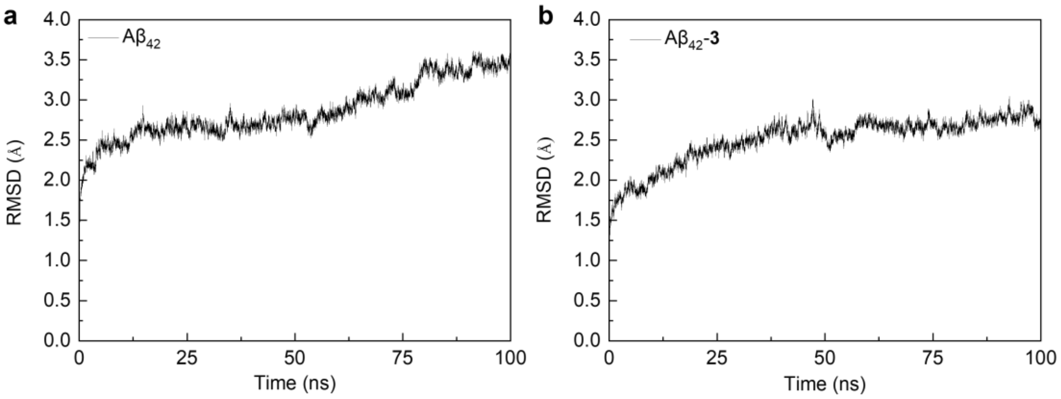

Supplementary Fig. 27 The RMSD value changes for Aβ_{42} protein (**a**) and the complex structures of Aβ_{42} with **3** (**b**) during 100 ns of MD simulations.

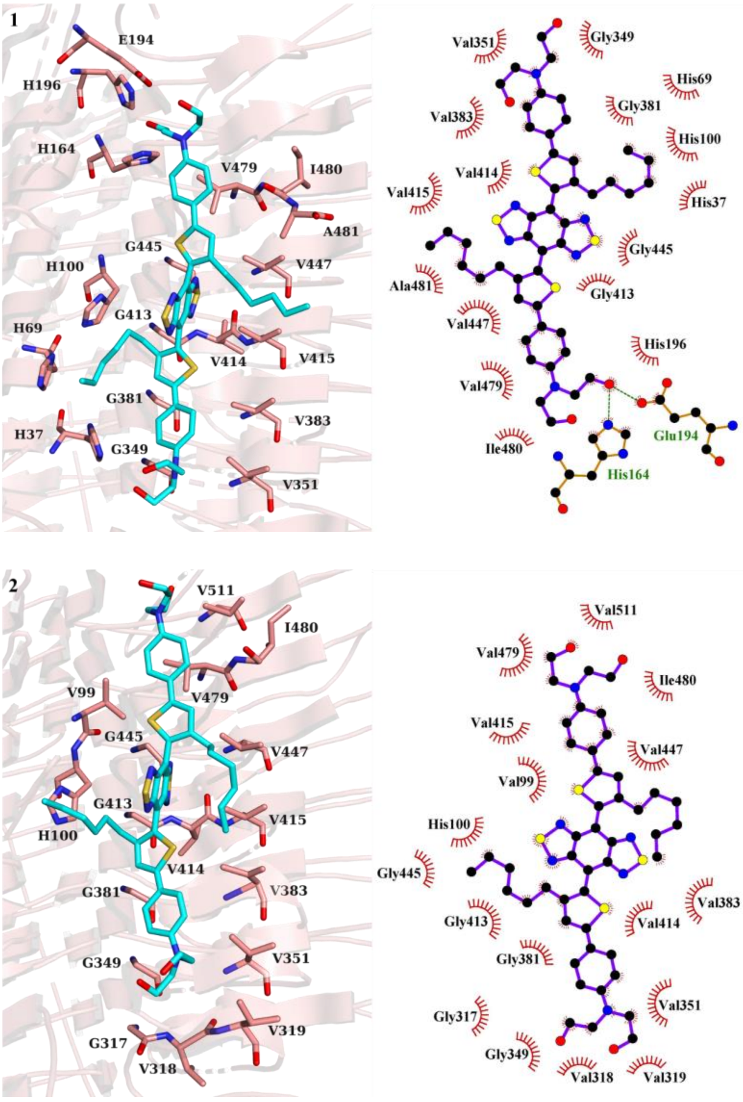

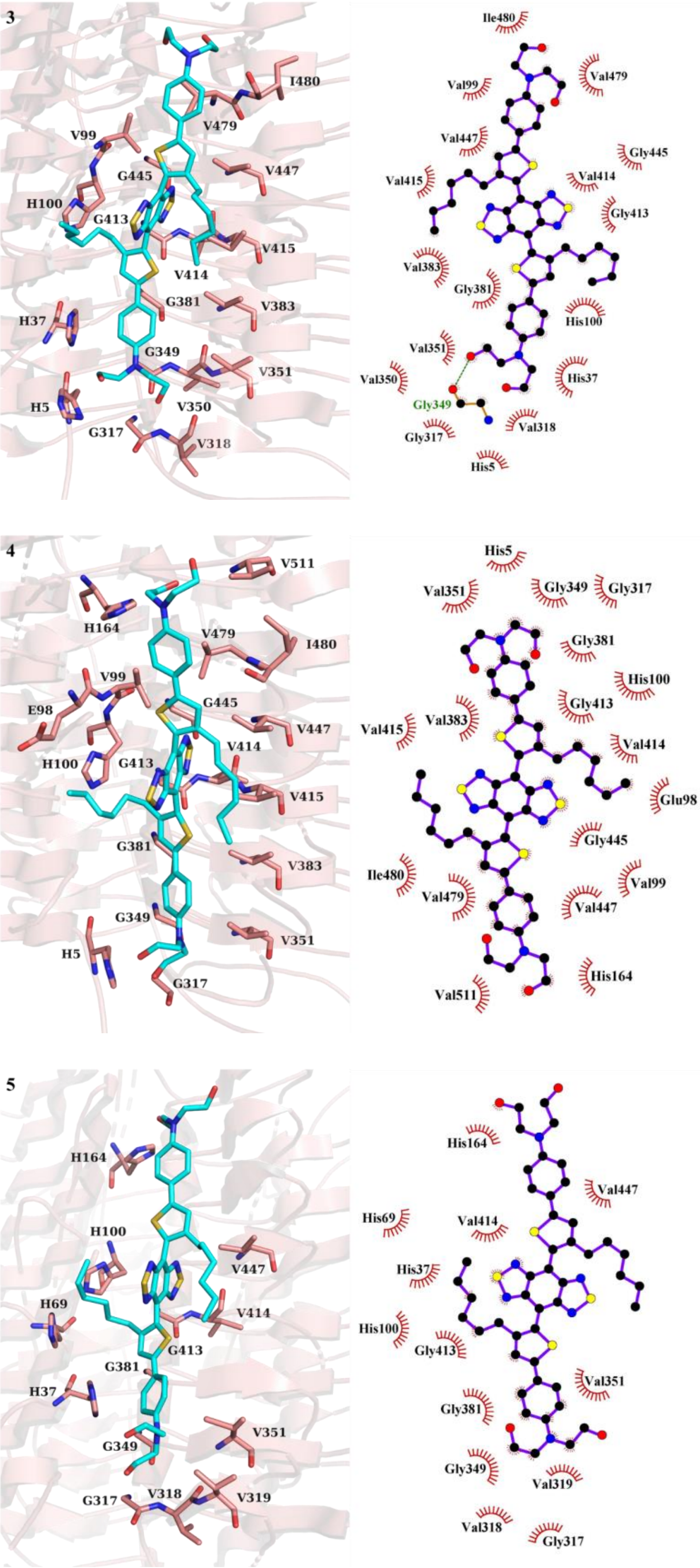
3
I480
V99
V479
G445
V447
H100
G413
V415
V414
G381
V383
H37
G349
V351
H5
V350
G317
V318
Ile480
Val99
Val479
Val447
Gly445
Val415
Val414
Gly413
Val383
Gly381
His100
Val351
Val350
His37
Gly349
Val318
Gly317
His5
4
V511
H164
V479
I480
V99
E98
G445
V447
H100
V414
G413
V415
G381
V383
G349
H5
V351
G317
His5
Val351
Gly349
Gly317
Gly381
His100
Gly413
Val415
Val383
Val414
Glu98
Gly445
Ile480
Val479
Val99
Val447
His164
Val511
5
H164
H100
V447
H69
V414
G413
G381
H37
V351
G349
G317
V318
V319
His164
Val447
His69
Val414
His37
His100
Gly413
Val351
Gly381
Gly349
Val319
Val318
Gly317

6

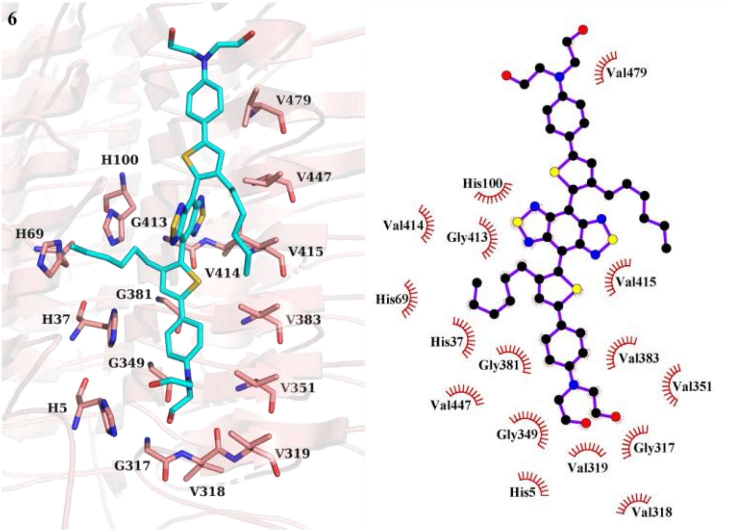
V479
H100
V447
G413
H69
V415
V414
G381
H37
V383
G349
V351
H5
G317
V319
V318
Val479
His100
Val414
Gly413
Val415
His69
His37
Gly381
Val383
Val351
Val447
Gly349
Gly317
Val319
His5
Val318

7

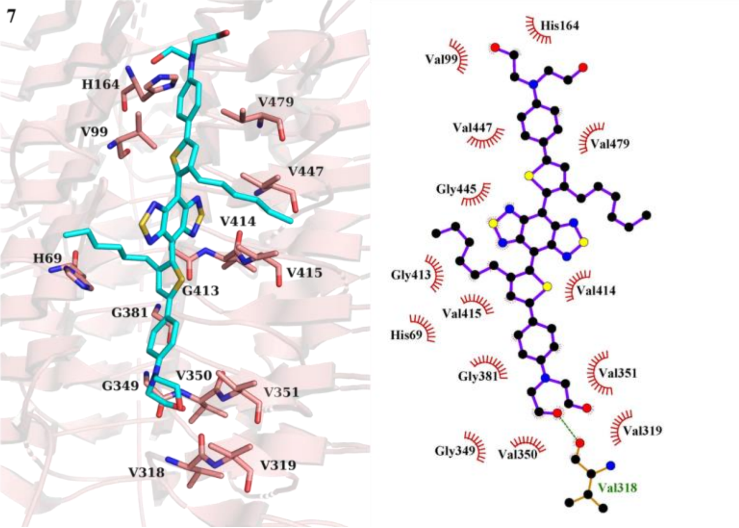
H164
V479
V99
V447
V414
H69
V415
G413
G381
V350
G349
V351
V318
V319
His164
Val99
Val447
Val479
Gly445
Gly413
Val414
Val415
His69
Val351
Gly381
Val319
Gly349
Val350
Val318

8

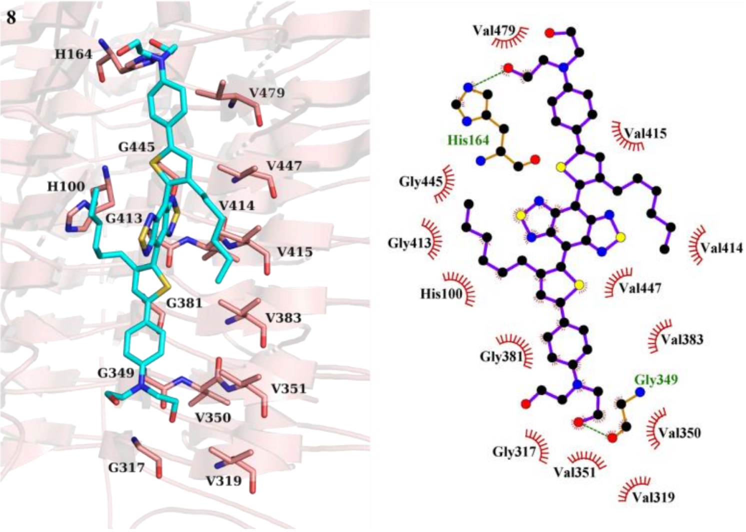
H164
V479
G445
V447
H100
V414
G413
V415
G381
V383
G349
V351
V350
G317
V319
Val479
His164
Val415
Gly445
Gly413
Val414
His100
Val447
Val383
Gly381
Gly349
Val350
Gly317
Val351
Val319

9

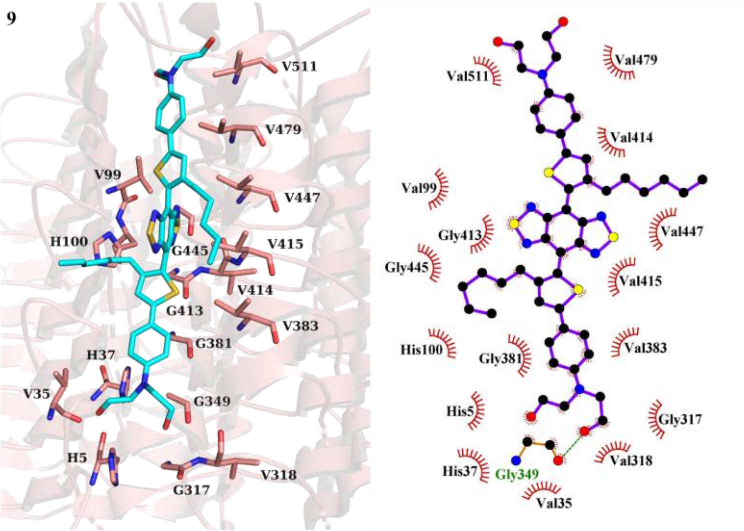

V511
V479
V99
V447
H100
G445
V415
V414
G413
V383
G381
H37
V35
G349
H5
V318
G317
Val479
Val511
Val414
Val99
Val447
Gly413
Gly445
Val415
His100
Gly381
Val383
His5
Gly317
Val318
His37
Gly349
Val35

10

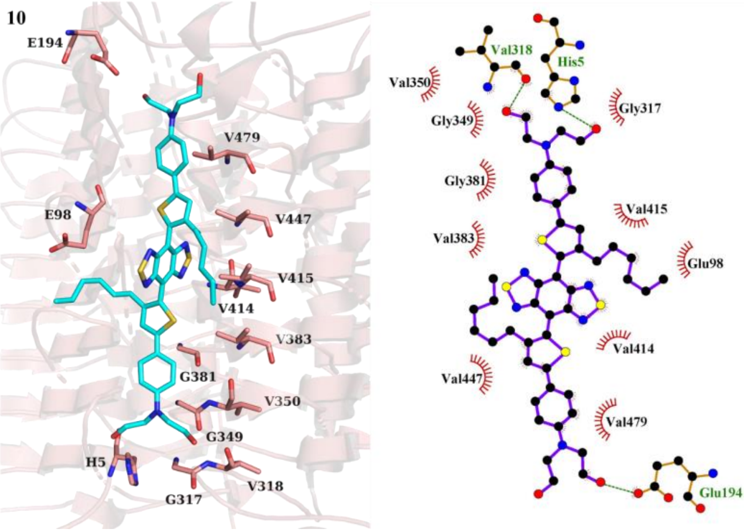

E194
V479
E98
V447
V415
V414
V383
G381
V350
G349
H5
V318
G317
Val318
His5
Val350
Gly349
Gly317
Gly381
Val415
Val383
Glu98
Val414
Val447
Val479
Glu194

11

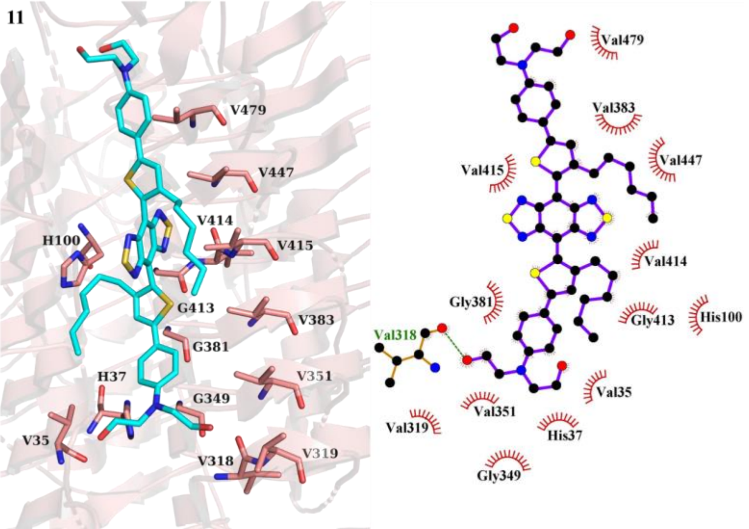

V479
V447
V414
H100
V415
G413
V383
G381
H37
V351
G349
V35
V318
V319
Val479
Val383
Val415
Val447
Val414
Gly381
Gly413
His100
Val318
Val35
Val351
Val319
His37
Gly349

12

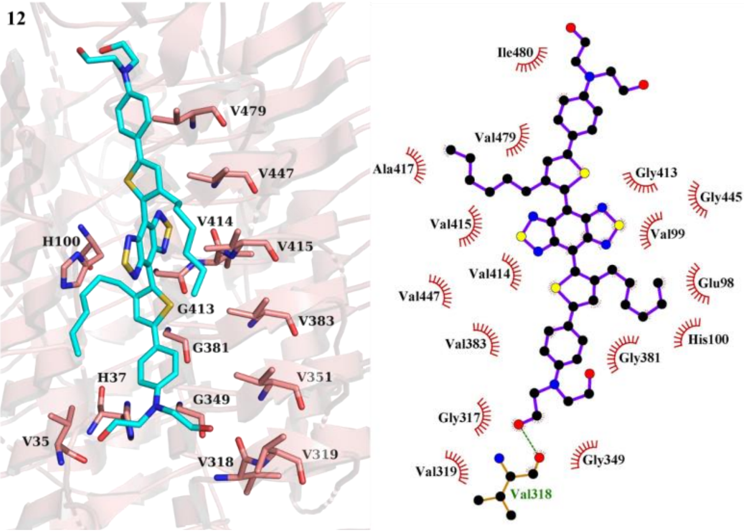
V479
V447
V414
H100
V415
G413
V383
G381
H37
V351
G349
V35
V318
V319
Ile480
Val479
Ala417
Gly413
Gly445
Val415
Val99
Val414
Glu98
Val447
His100
Val383
Gly381
Gly317
Gly349
Val319
Val318

13

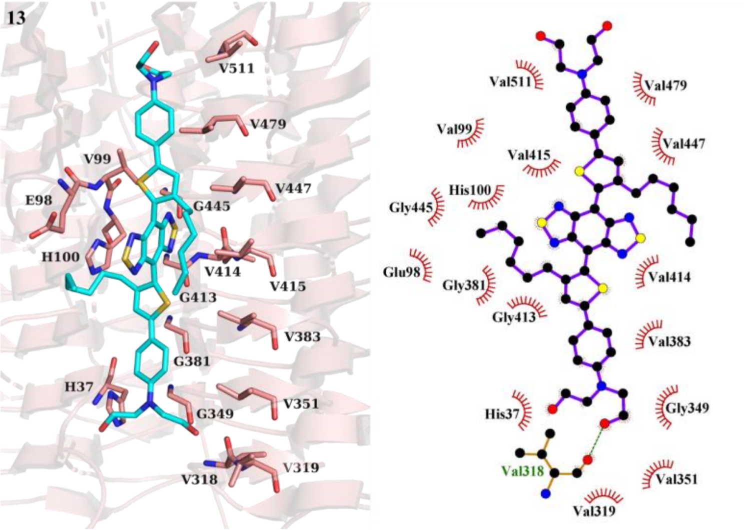
V511
V479
V99
V447
E98
G445
H100
V414
V415
G413
V383
G381
H37
G349
V351
V318
V319
Val511
Val479
Val99
Val447
Val415
His100
Gly445
Val414
Glu98
Gly381
Gly413
Val383
His37
Gly349
Val318
Val351
Val319

14

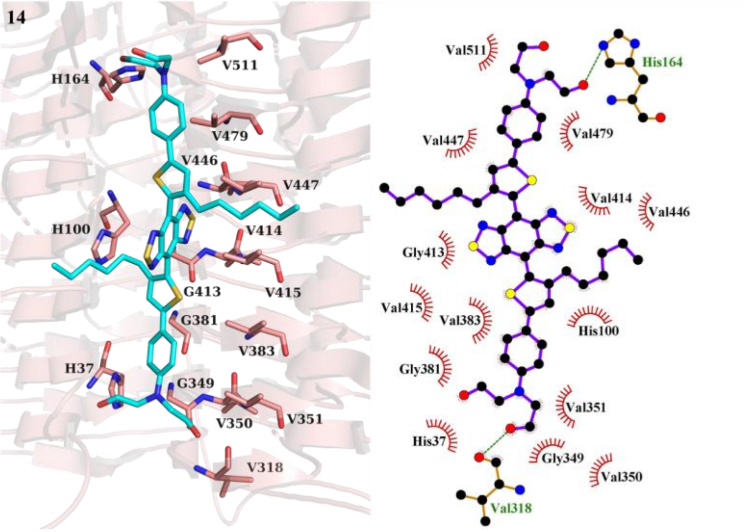
H164
V511
V479
V446
V447
H100
V414
G413
V415
G381
V383
H37
G349
V350
V351
V318
Val511
His164
Val447
Val479
Val414
Val446
Gly413
Val415
Val383
His100
Gly381
Val351
His37
Gly349
Val350
Val318

15

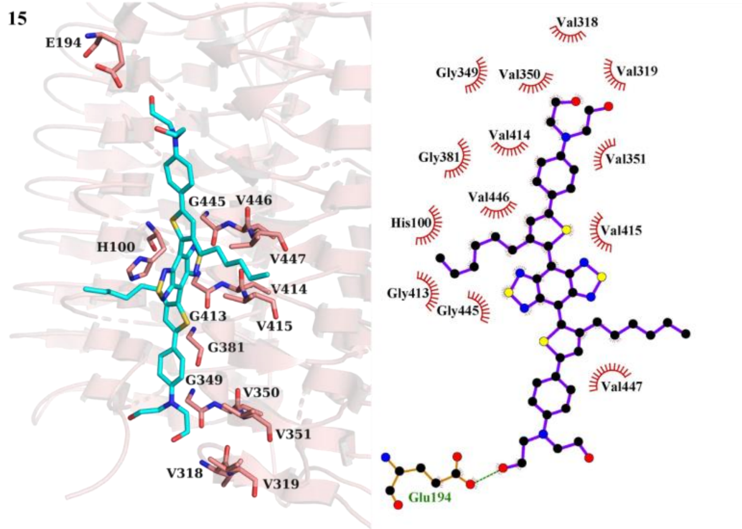
E194
G445
V446
H100
V447
V414
G413
V415
G381
G349
V350
V351
V318
V319
Val318
Gly349
Val350
Val319
Val414
Gly381
Val351
Val446
His100
Val415
Gly413
Gly445
Val447
Glu194

16

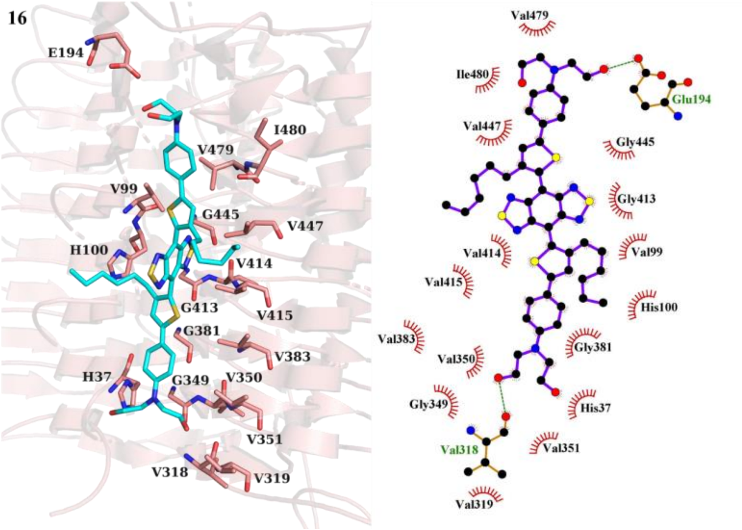
E194
I480
V479
V99
G445
V447
H100
V414
G413
V415
G381
V383
H37
G349
V350
V351
V318
V319
Val479
Ile480
Glu194
Val447
Gly445
Gly413
Val414
Val99
Val415
His100
Val383
Gly381
Val350
Gly349
His37
Val318
Val351
Val319

17

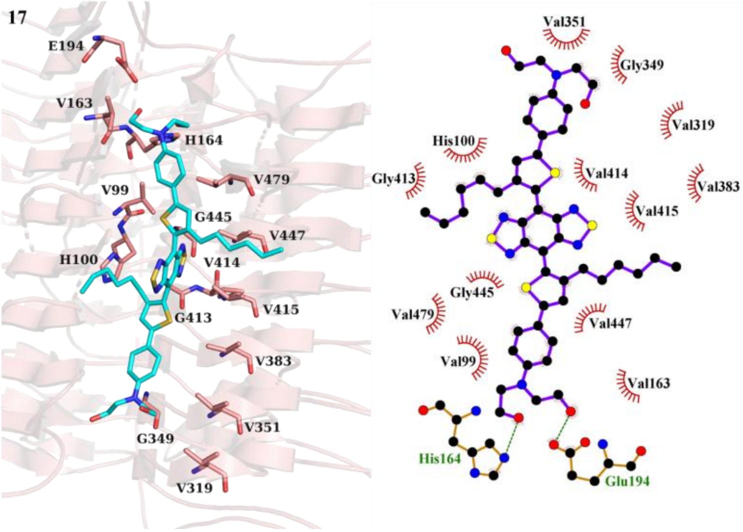
E194
V163
H164
V479
V99
G445
V447
H100
V414
G413
V415
V383
G349
V351
V319
Val351
Gly349
Val319
His100
Val414
Val383
Gly413
Val415
Gly445
Val479
Val447
Val99
Val163
His164
Glu194

18

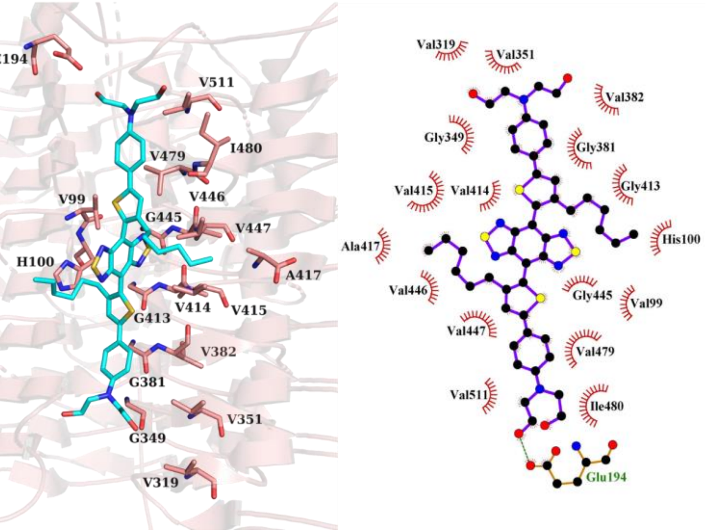
E194
V511
I480
V479
V99
V446
G445
V447
H100
A417
V414
V415
G413
V382
G381
V351
G349
V319
Val319
Val351
Val382
Gly349
Gly381
Val415
Val414
Gly413
Ala417
His100
Val446
Gly445
Val99
Val447
Val479
Val511
Ile480
Glu194

19

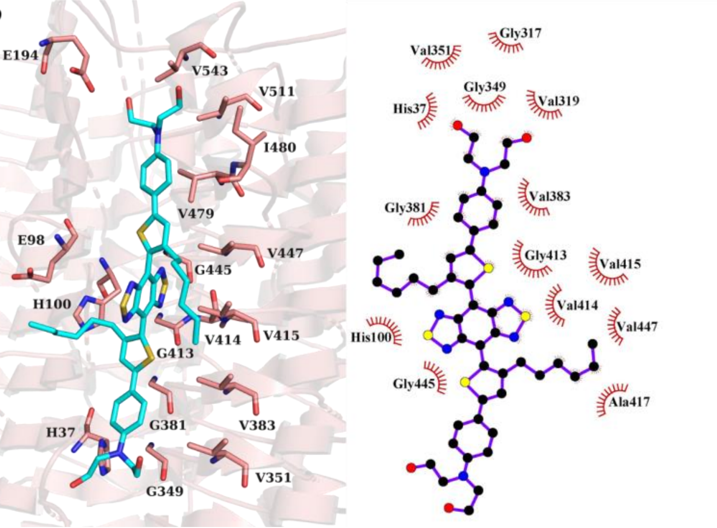
E194
V543
V511
I480
V479
E98
V447
G445
H100
V414
V415
G413
H37
G381
V383
V351
G349
Gly317
Val351
Gly349
His37
Val319
Val383
Gly381
Gly413
Val415
Val414
His100
Val447
Gly445
Ala417

20

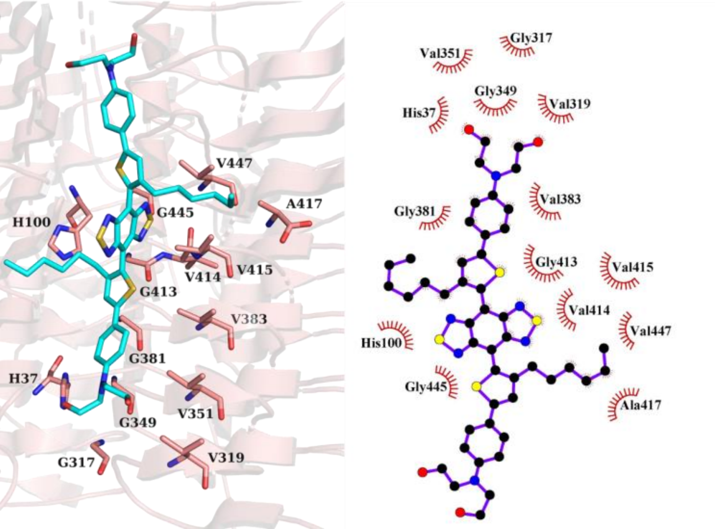
V447
A417
G445
H100
V414
V415
G413
V383
G381
H37
V351
G349
G317
V319
Gly317
Val351
Gly349
His37
Val319
Val383
Gly381
Gly413
Val415
Val414
His100
Val447
Gly445
Ala417

21

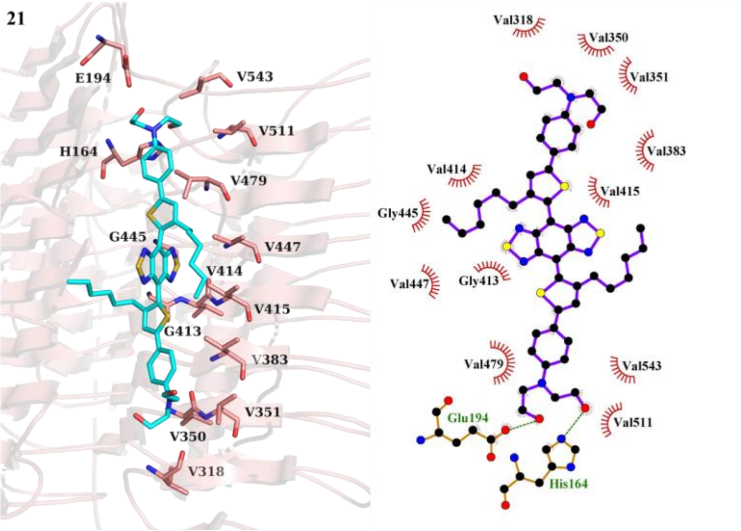
E194
V543
V511
H164
V479
G445
V447
V414
V415
G413
V383
V351
V350
V318
Val318
Val350
Val351
Val383
Val414
Val415
Gly445
Val447
Gly413
Val479
Val543
Glu194
Val511
His164

22

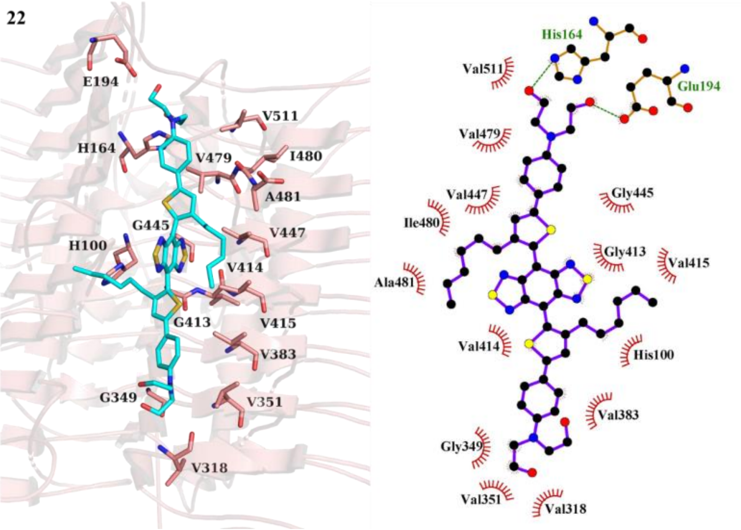
E194
V511
H164
V479
I480
A481
G445
V447
H100
V414
G413
V415
V383
G349
V351
V318
His164
Val511
Glu194
Val479
Val447
Gly445
Ile480
Gly413
Val415
Ala481
Val414
His100
Val383
Gly349
Val351
Val318

23

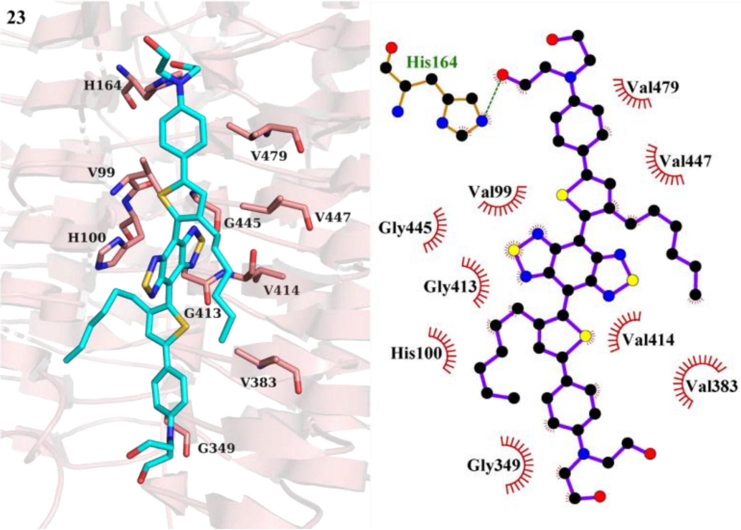
H164
V479
V99
G445
V447
H100
V414
G413
V383
G349
His164
Val479
Val447
Val99
Gly445
Gly413
Val414
His100
Val383
Gly349

24

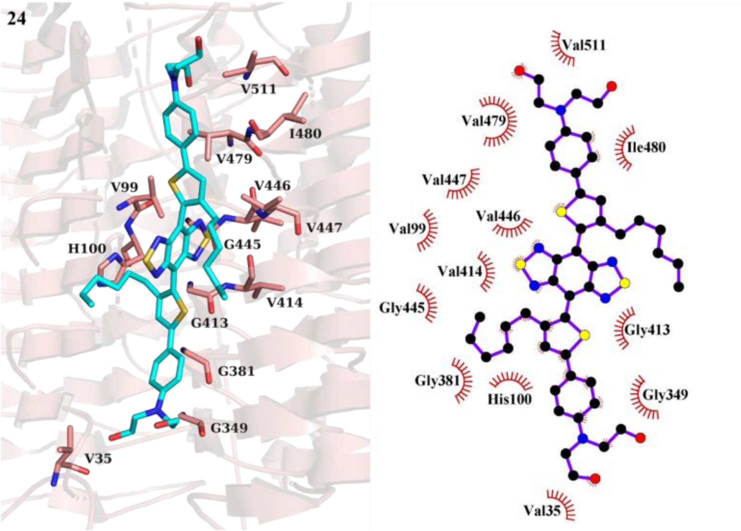
V511
I480
V479
V446
V99
V447
H100
G445
V414
G413
G381
G349
V35
Val511
Val479
Ile480
Val447
Val446
Val99
Val414
Gly445
Gly413
Gly381
His100
Gly349
Val35

25

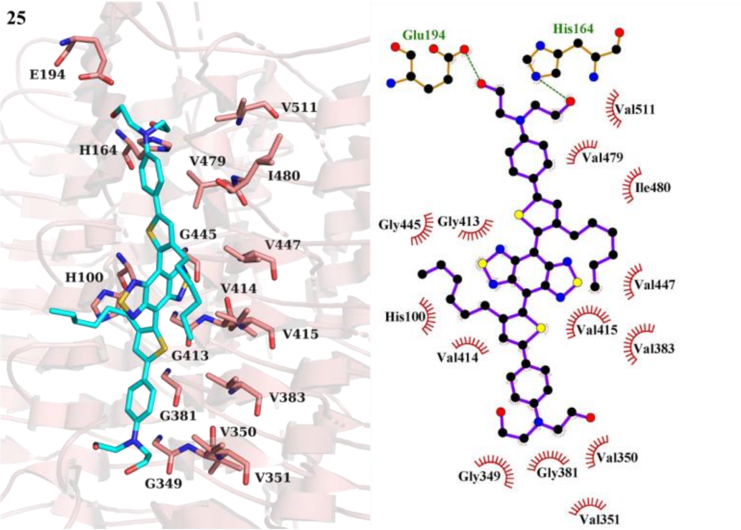
E194
V511
H164
V479
I480
G445
V447
H100
V414
V415
G413
V383
G381
V350
V351
G349
Glu194
His164
Val511
Val479
Ile480
Gly445
Gly413
Val447
His100
Val415
Val383
Val414
Val350
Gly349
Gly381
Val351

26

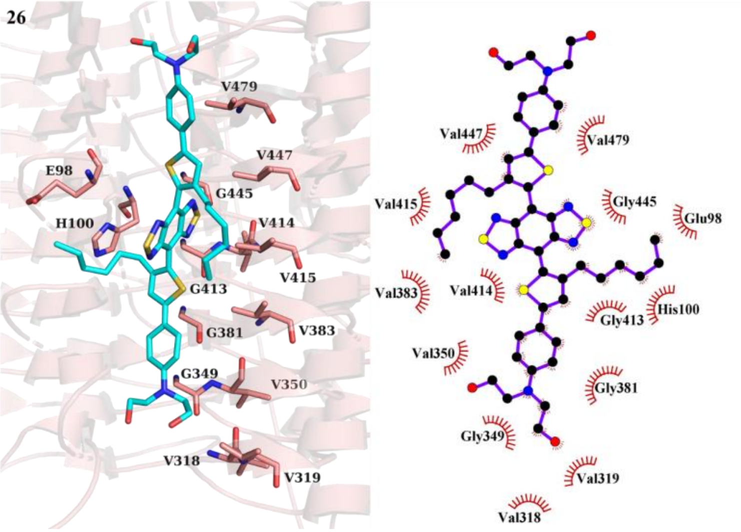
V479
V447
E98
G445
H100
V414
V415
G413
G381
V383
G349
V350
V318
V319
Val447
Val479
Val415
Gly445
Glu98
Val383
Val414
Gly413
His100
Val350
Gly381
Gly349
Val319
Val318

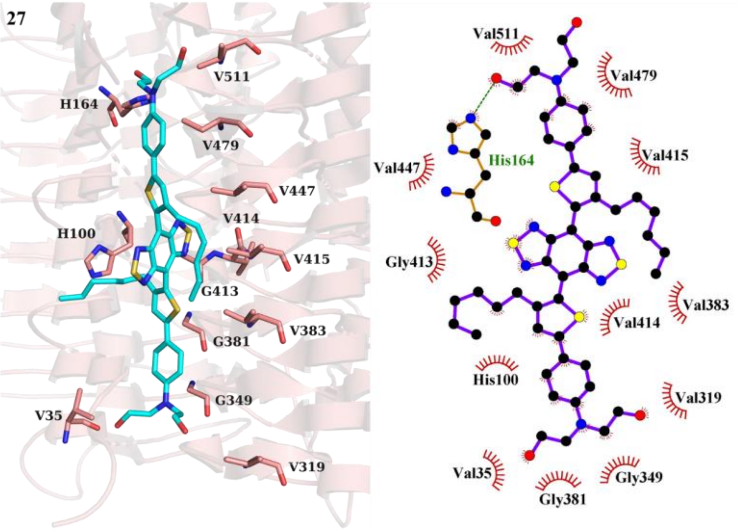
27
V511
H164
V479
V447
V414
H100
V415
G413
V383
G381
G349
V35
V319
Val511
Val479
His164
Val447
Val415
Gly413
Val414
Val383
His100
Val319
Val35
Gly381
Gly349

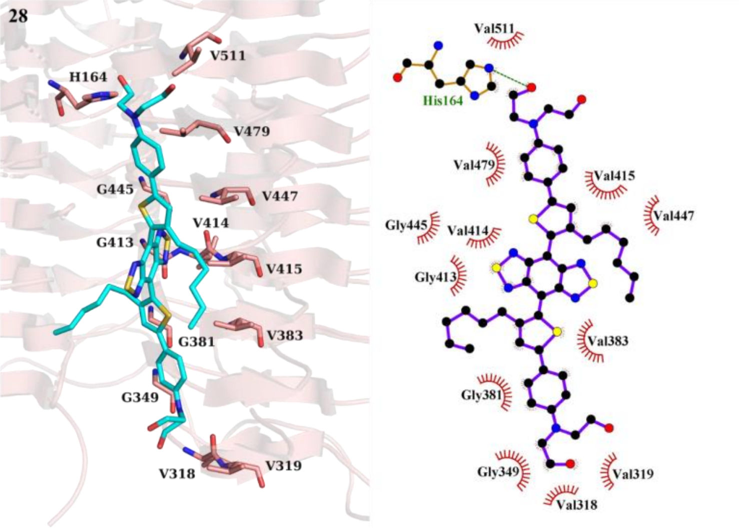
28
V511
H164
V479
G445
V447
V414
G413
V415
G381
V383
G349
V318
V319
Val511
His164
Val479
Val415
Val447
Gly445
Val414
Gly413
Val383
Gly381
Gly349
Val319
Val318

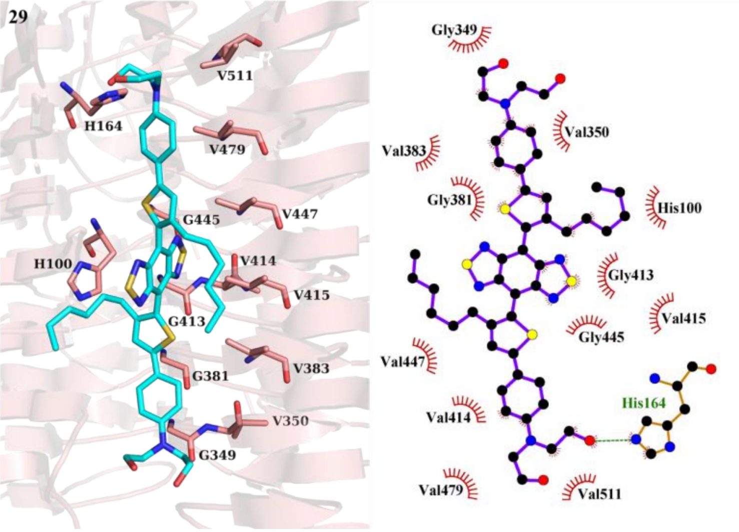
29
V511
H164
V479
V447
G445
H100
V414
V415
G413
G381
V383
V350
G349
Gly349
Val350
Val383
Gly381
His100
Gly413
Val415
Gly445
Val447
His164
Val414
Val479
Val511

30

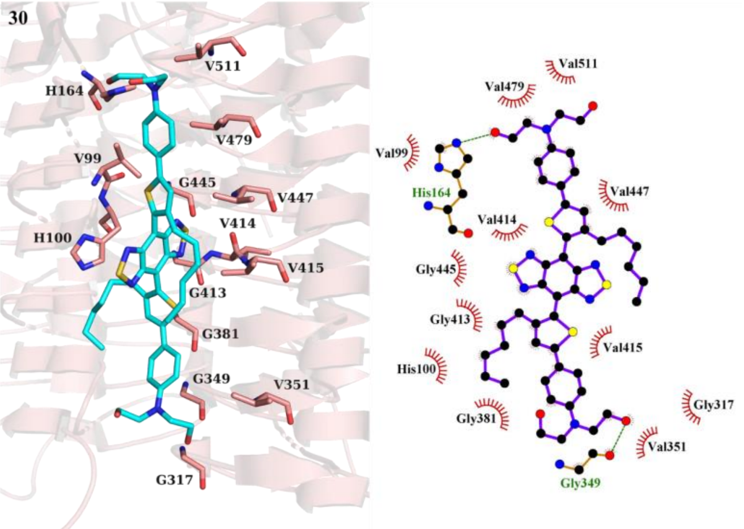
H164
V511
V479
V99
G445
V447
V414
H100
V415
G413
G381
G349
V351
G317
Val511
Val479
Val99
His164
Val447
Val414
Gly445
Gly413
Val415
His100
Gly317
Gly381
Val351
Gly349

31

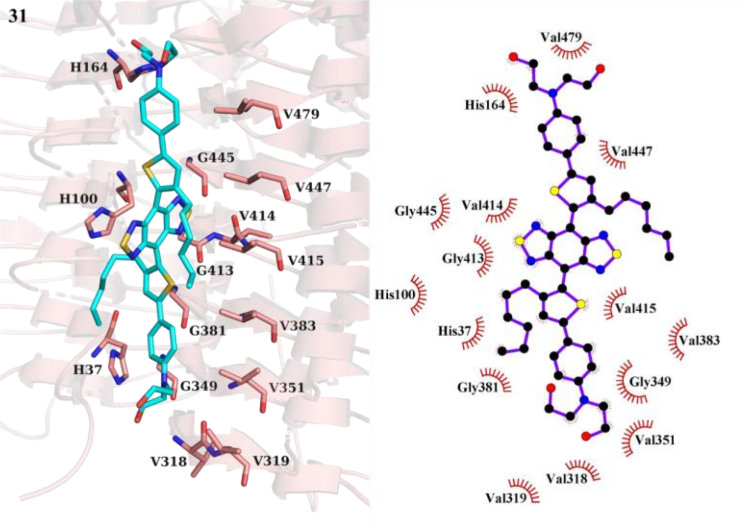
H164
V479
G445
V447
H100
V414
V415
G413
G381
V383
H37
G349
V351
V318
V319
Val479
His164
Val447
Gly445
Val414
Gly413
His100
Val415
His37
Val383
Gly381
Gly349
Val351
Val318
Val319

32

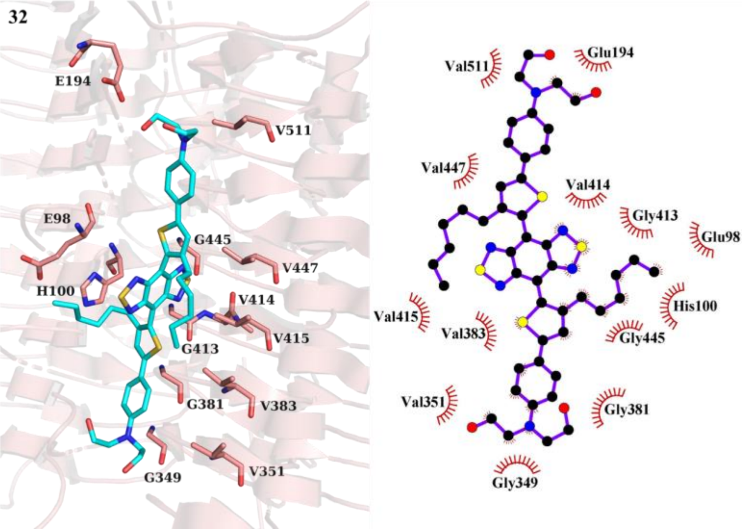
E194
V511
E98
G445
V447
H100
V414
V415
G413
G381
V383
G349
V351
Val511
Glu194
Val447
Val414
Gly413
Glu98
His100
Val415
Val383
Gly445
Val351
Gly381
Gly349

33

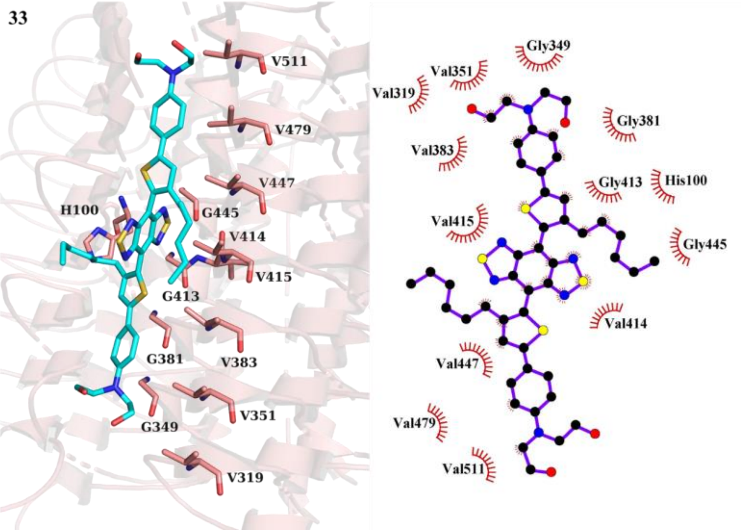
V511
V479
V447
H100
G445
V414
V415
G413
G381
V383
V351
G349
V319
Gly349
Val351
Val319
Gly381
Val383
Gly413
His100
Val415
Gly445
Val414
Val447
Val479
Val511

34

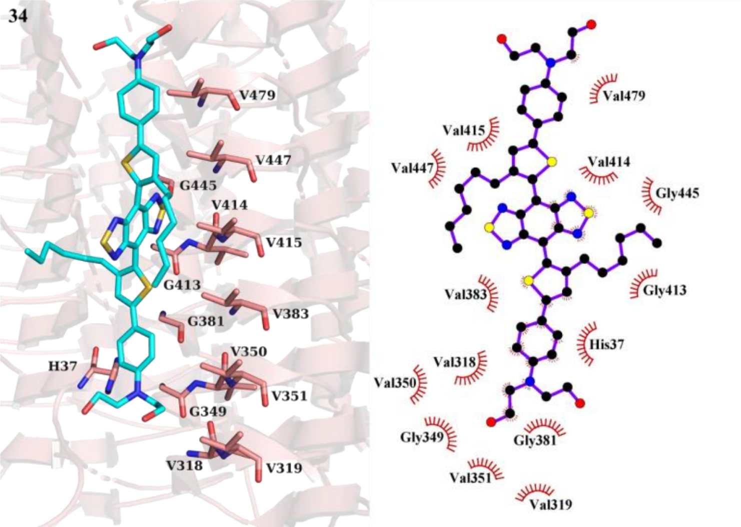
V479
V447
G445
V414
V415
G413
V383
G381
H37
V350
V351
G349
V318
V319
Val479
Val415
Val447
Val414
Gly445
Val383
Gly413
His37
Val318
Val350
Gly349
Gly381
Val351
Val319

35

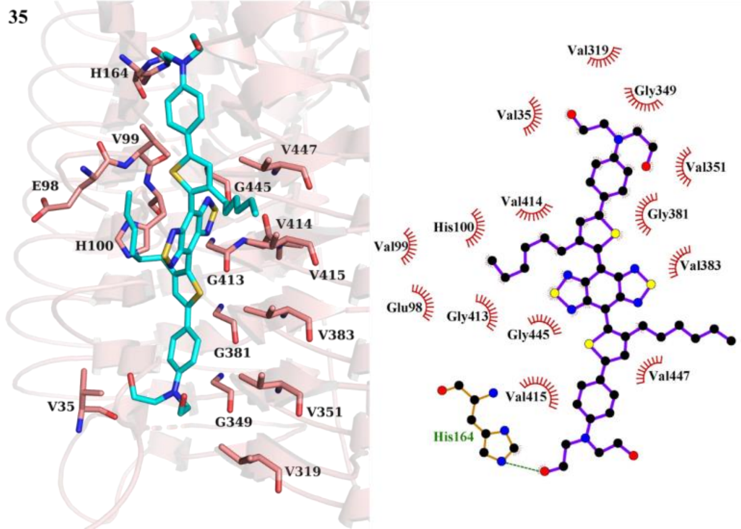
H164
V99
E98
V447
G445
V414
H100
V415
G413
V383
G381
V35
G349
V351
V319
Val319
Gly349
Val35
Val351
Val414
Gly381
His100
Val99
Val383
Glu98
Gly413
Gly445
Val447
Val415
His164

36

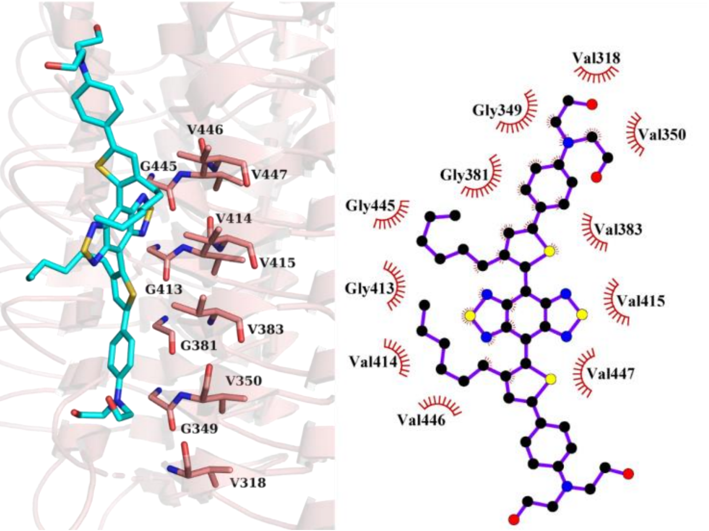

V446
G445
V447
V414
V415
G413
V383
G381
V350
G349
V318
Val318
Gly349
Val350
Gly381
Gly445
Val383
Gly413
Val415
Val414
Val447
Val446

37

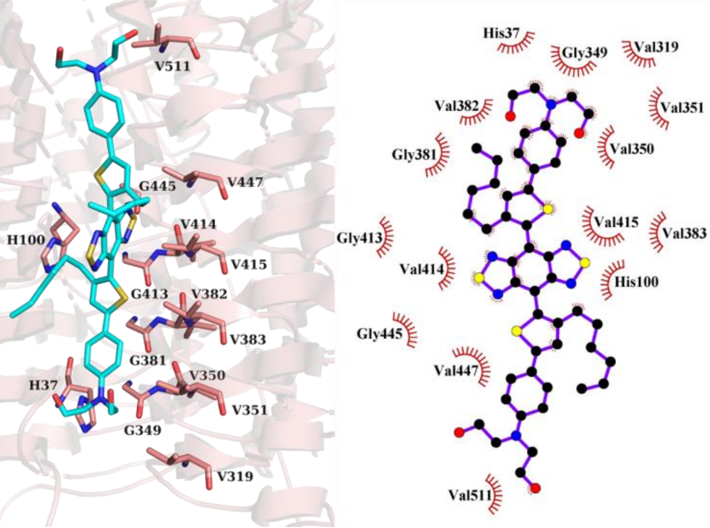

V511
G445
V447
V414
H100
V415
G413
V382
V383
G381
H37
V350
V351
G349
V319
His37
Gly349
Val319
Val382
Val351
Gly381
Val350
Val415
Val383
Gly413
Val414
His100
Gly445
Val447
Val511

38

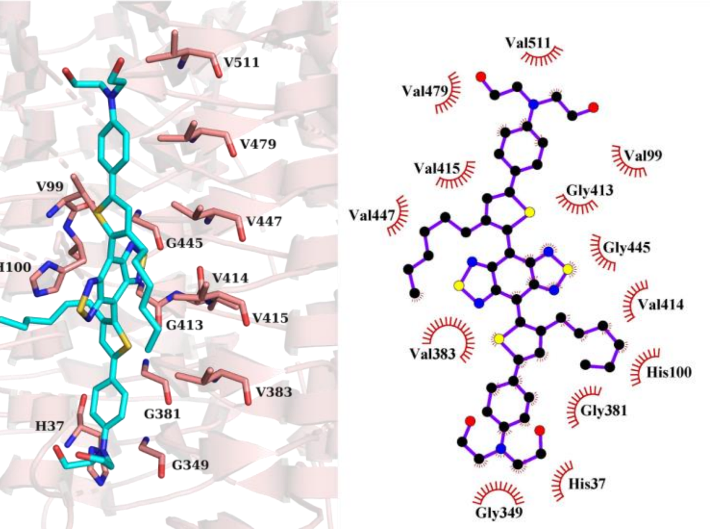

V511
V479
V99
V447
G445
H100
V414
V415
G413
V383
G381
H37
G349
Val511
Val479
Val99
Val415
Gly413
Val447
Gly445
Val414
Val383
His100
Gly381
His37
Gly349

39

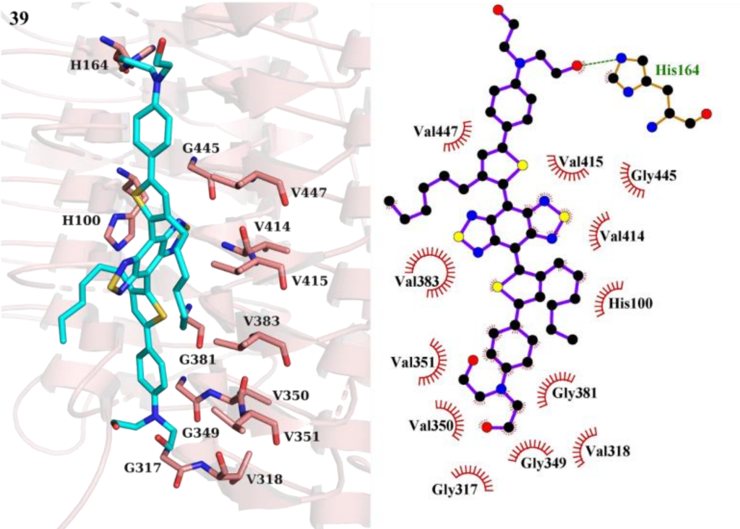
H164
G445
V447
H100
V414
V415
V383
G381
V350
G349
V351
G317
V318
His164
Val447
Val415
Gly445
Val414
Val383
His100
Val351
Gly381
Val350
Val318
Gly349
Gly317

40

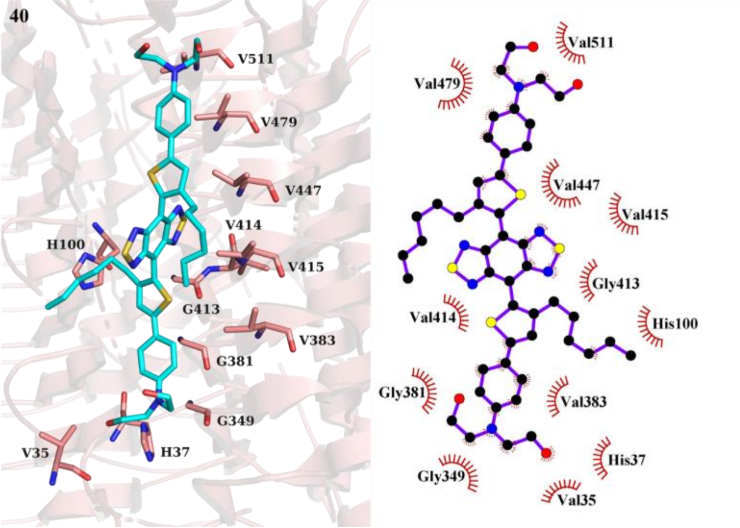
V511
V479
V447
V414
H100
V415
G413
V383
G381
G349
V35
H37
Val511
Val479
Val447
Val415
Gly413
Val414
His100
Gly381
Val383
His37
Gly349
Val35

41

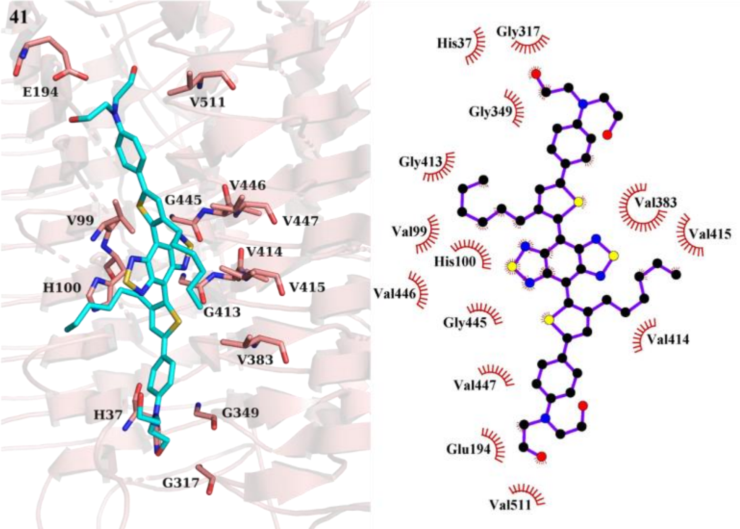
E194
V511
V446
G445
V447
V99
V414
H100
V415
G413
V383
H37
G349
G317
His37
Gly317
Gly349
Gly413
Val383
Val99
Val415
His100
Val446
Gly445
Val414
Val447
Glu194
Val511

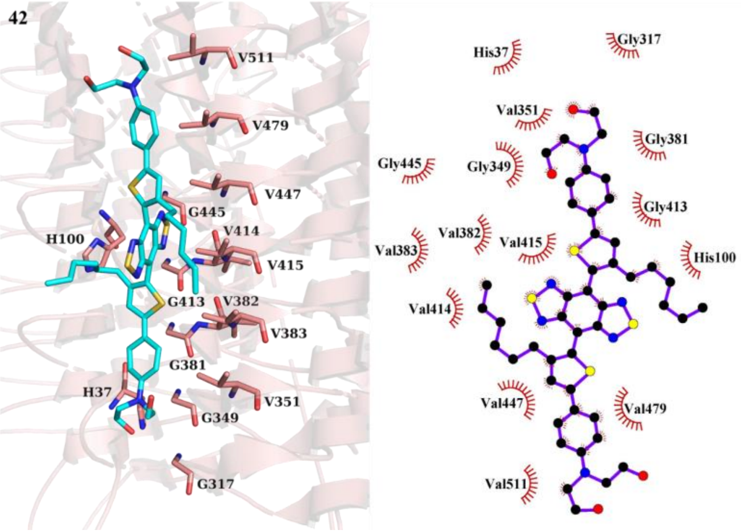
42
V511
V479
V447
G445
V414
H100
V415
G413
V382
V383
G381
H37
V351
G349
G317
His37
Gly317
Val351
Gly381
Gly445
Gly349
Gly413
Val382
Val415
Val383
His100
Val414
Val447
Val479
Val511

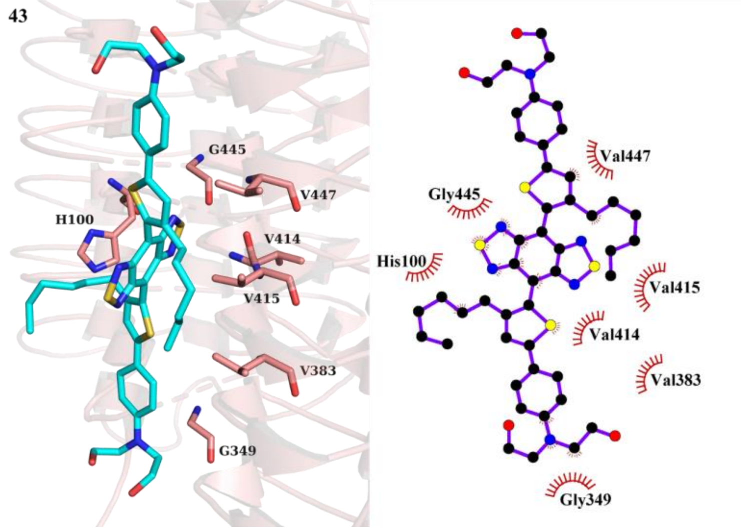
43
G445
V447
H100
V414
V415
V383
G349
Val447
Gly445
His100
Val415
Val414
Val383
Gly349

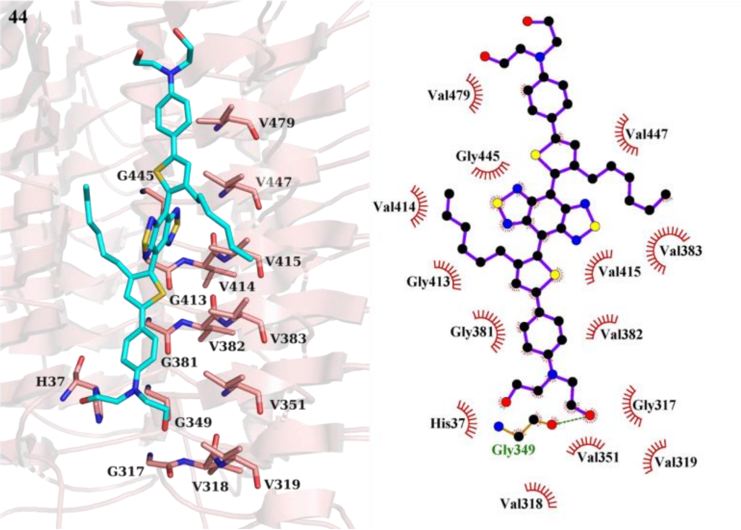
44
V479
G445
V447
V415
V414
G413
V382
V383
G381
H37
V351
G349
G317
V318
V319
Val479
Val447
Gly445
Val414
Val383
Gly413
Val415
Gly381
Val382
His37
Gly317
Gly349
Val351
Val319
Val318

45

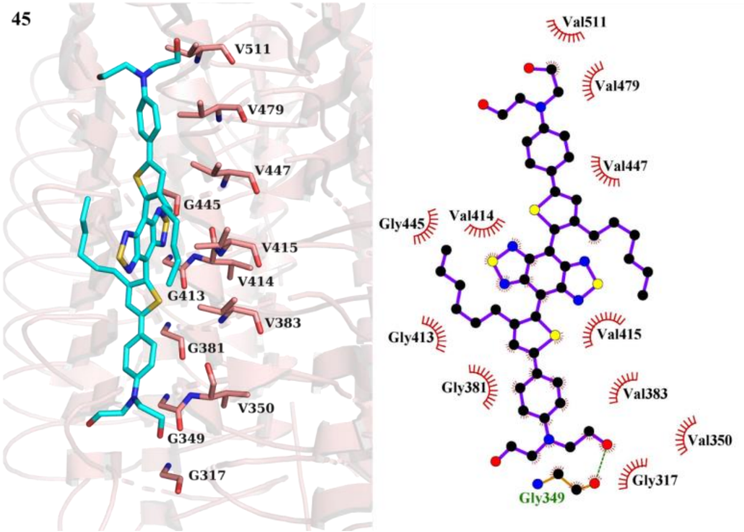

V511
V479
V447
G445
V415
V414
G413
V383
G381
V350
G349
G317
Val511
Val479
Val447
Gly445
Val414
Gly413
Val415
Gly381
Val383
Val350
Gly317
Gly349

46

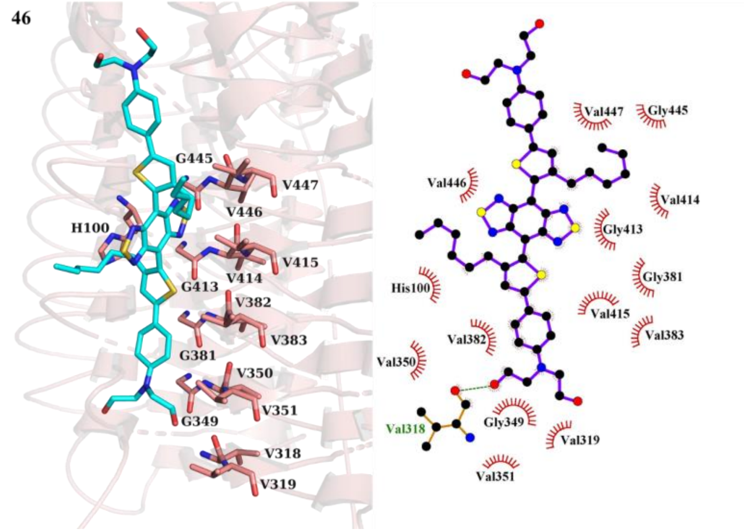

G445
V447
V446
H100
V415
V414
G413
V382
V383
G381
V350
V351
G349
V318
V319
Val447
Gly445
Val446
Val414
Gly413
His100
Gly381
Val415
Val382
Val383
Val350
Val318
Gly349
Val319
Val351

47

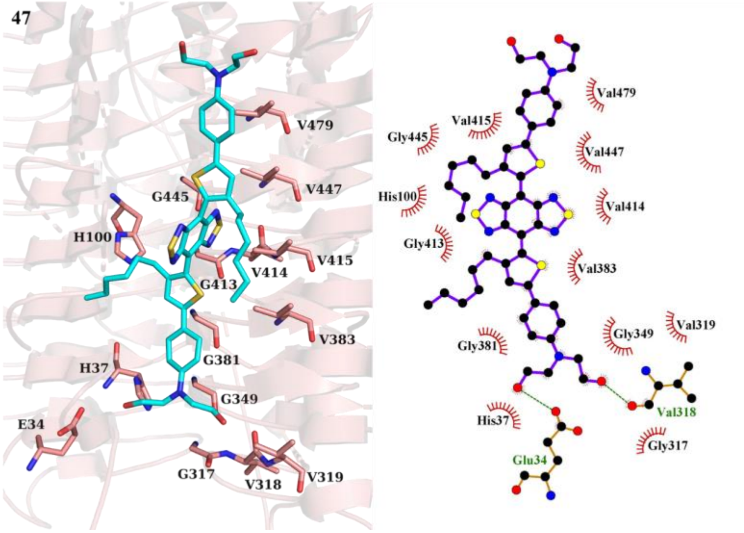

V479
V447
G445
H100
V414
V415
G413
V383
G381
H37
G349
E34
G317
V318
V319
Val479
Val415
Gly445
Val447
His100
Val414
Gly413
Val383
Gly349
Val319
Gly381
His37
Val318
Gly317
Glu34

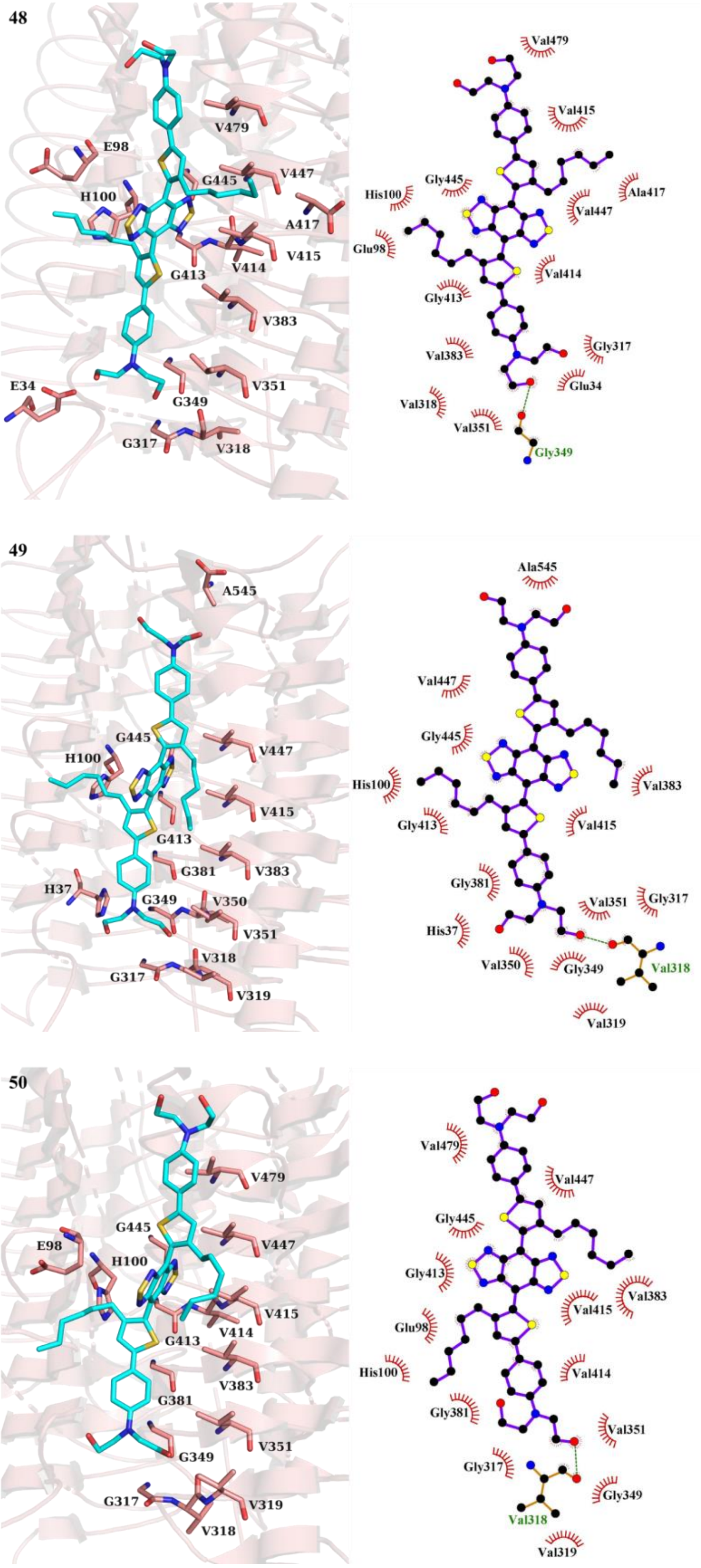

Supplementary Fig. 28 The key residues of the $A\beta_{42}$ protein that are close to **3** in different snapshots with a time gap of 2 ns, are shown in the three-dimensional diagrams (on the left) and top-view (on the right).

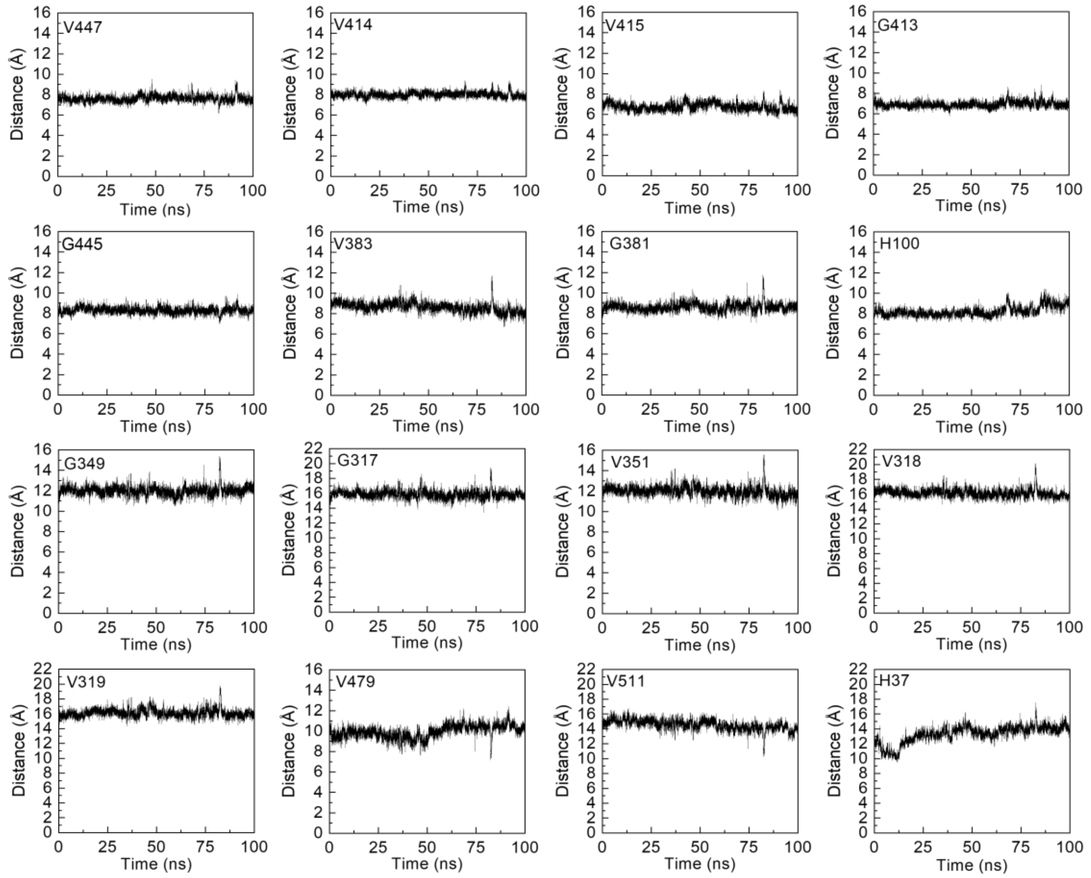

Supplementary Fig. 29 The distance changes of 16 active-site residues of Aβ protein to the mass center of **3** in 100 ns MD simulations.

Supplementary Table 1. The frequencies of residues in the $A\beta_{42}$ protein that are active with **3**.

Number	Type	Sum
1	Val447	50
2	Gly349	49
3	Val414	49
4	Gly413	47
5	Val415	47
6	Val383	44
7	Gly381	43
8	His100	42
9	Gly445	42
10	Val479	38
11	Val351	34
12	Val318	27
13	Val319	26
14	Val511	23
15	His37	21
16	Gly317	20

Supplementary Table 2. DFT-computed Cartesian coordinates.

3 in S_0 state

E = -4008.819133 Hartree

C	-7.04172300	-1.55342400	-1.32726400
C	-6.76666100	-0.43945800	-0.51510600
C	-7.86665000	0.35100600	-0.13658000
C	-9.16126500	0.04790200	-0.53207100
C	-9.43712700	-1.06930200	-1.35716200
C	-8.33036700	-1.86543000	-1.73697400
H	-6.23020400	-2.21070600	-1.62866600
H	-7.70252700	1.23852800	0.46788500
H	-9.95995700	0.70193000	-0.20388900
H	-8.46887700	-2.74690400	-2.35085000
N	-10.72959600	-1.36000100	-1.78544800
C	-11.86462400	-0.57929100	-1.31576700
H	-11.63675500	0.49174400	-1.35308900
H	-12.70347700	-0.73664400	-1.99958400
C	-10.99769400	-2.53127000	-2.60671700
H	-10.23522900	-2.63392300	-3.38635200
H	-11.94863300	-2.38410700	-3.12688500
C	-11.06845700	-3.84167800	-1.81510800
H	-11.91923500	-3.80285400	-1.11548800
H	-10.15319500	-3.96119600	-1.21557400
C	-12.31587800	-0.93376000	0.10533100
H	-12.68000500	-1.97374200	0.12664300
H	-11.45822200	-0.86987400	0.79211800
O	-13.34073000	-0.00845400	0.44523000
H	-13.64015000	-0.20339000	1.34549500
O	-11.21920100	-4.88258600	-2.77178000
H	-11.25072400	-5.72776000	-2.29973900
C	-5.41583500	-0.10839000	-0.06734200
C	-5.04084600	0.58761500	1.06307100
S	-4.00149100	-0.59016000	-0.97104600
C	-3.63841400	0.75299400	1.22753300
H	-5.76395900	0.93749000	1.79142800
C	-2.91977400	0.17333000	0.18976800
C	-3.03342000	1.38635500	2.45692800
C	-3.58479200	0.81580300	3.77585000
H	-3.19631700	2.47238400	2.42962600
H	-1.94860900	1.24839500	2.44490300
C	-2.90770800	1.43082700	5.00560800
H	-4.66813100	0.98591400	3.83894200
H	-3.44615900	-0.27402300	3.78265600
C	-3.44385900	0.88076200	6.33196600
H	-1.82331600	1.25368000	4.95016700
H	-3.03784100	2.52328300	4.98462800
H	-4.52820000	1.05958500	6.38903600
H	-3.31560500	-0.21182400	6.35105100
C	-2.76542700	1.49135200	7.56402200
C	-3.30865000	0.93491700	8.88392100
H	-2.89392100	2.58306400	7.54362700
H	-1.68243900	1.31143100	7.50629200
H	-2.80818100	1.38649500	9.74825000
H	-4.38412800	1.12952100	8.98058400
H	-3.16356900	-0.15100000	8.94285500
C	-1.48621800	0.19152800	-0.05389400
C	-0.74385600	-0.95451100	-0.43755300
C	-0.71205700	1.37564800	0.03448100
C	0.70394700	-0.92181500	-0.69984400
C	0.74254700	1.40358900	-0.20244200
C	1.48414600	0.26112700	-0.59912400

N	-1.27500900	-2.18579100	-0.59143600
N	1.19469500	-2.12671400	-1.05842800
N	-1.21440100	2.59263300	0.33915600
N	1.27586600	2.62773900	-0.00001400
S	-0.05592600	-3.17056100	-1.03873800
S	0.04470700	3.62436100	0.37796600
C	2.91589400	0.29247000	-0.85480200
S	3.96128200	-0.93879600	-0.14821500
C	5.04999200	0.89393900	-1.58137300
H	5.77829700	1.46395600	-2.14881200
C	3.66281600	1.19893500	-1.59810800
C	3.11637400	2.33264900	-2.42831900
C	3.59384100	3.71688100	-1.95205800
H	3.43221100	2.18644000	-3.47165400
H	2.02374400	2.30558700	-2.43163600
H	3.40499100	3.80010300	-0.87691300
H	4.68142100	3.79729800	-2.09097900
C	2.89316600	4.86331100	-2.68709900
C	3.35959000	6.24968700	-2.22993100
H	1.80768800	4.77586200	-2.52873100
H	3.05182800	4.76276900	-3.77145000
C	2.64222800	7.40158700	-2.94359700
H	3.20604100	6.34349800	-1.14427100
H	4.44425200	6.34181300	-2.39098600
C	3.11366900	8.78226200	-2.47636800
H	2.79631900	7.30695000	-4.02802500
H	1.55888400	7.30781500	-2.78137600
H	2.58596200	9.58748600	-3.00043800
H	2.94156000	8.91394900	-1.40077500
H	4.18799600	8.91347800	-2.65655500
C	5.39306800	-0.22045000	-0.84381200
C	9.35982400	-1.85364100	-0.21728400
C	9.14426900	-0.48132600	-0.50576600
C	7.87007300	0.02762500	-0.70204700
C	6.72258000	-0.78320700	-0.62626900
C	6.93176800	-2.14125400	-0.33110200
C	8.20072200	-2.66431600	-0.12641300
H	9.97725100	0.20745800	-0.56912300
H	7.75887600	1.09118800	-0.89366200
H	6.08034200	-2.81496000	-0.27702100
H	8.28227700	-3.72045100	0.09393100
N	10.63142500	-2.38045600	-0.04043600
C	11.78322200	-1.49409900	0.05147200
C	11.91017400	-0.77402600	1.39397400
H	11.73545500	-0.75041100	-0.75142600
H	12.68353600	-2.07588500	-0.14496100
H	12.73504400	-0.04402600	1.33214400
H	10.98309300	-0.22270700	1.60664000
C	12.15192700	-4.33295100	0.51830800
C	10.74355300	-3.78627700	0.35544100
H	12.04528900	-5.37002700	0.87522100
H	12.69518300	-3.76818000	1.28539700
H	10.24578700	-4.40221600	-0.40478700
H	10.22297100	-3.95347700	1.31133100
O	12.16290200	-1.75482500	2.39881700
H	12.05391700	-1.33688800	3.26548500
O	12.81443900	-4.29499000	-0.74646200
H	13.72151700	-4.60980700	-0.61458000

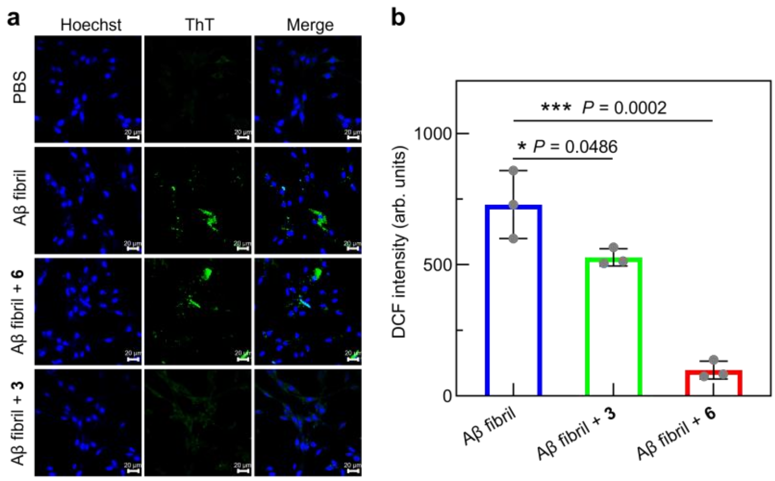

Supplementary Fig. 30 a Confocal images of PC12 cells incubated with PBS, Aβ fibril, Aβ fibril with **6**, and Aβ fibril with **3** for 48 h. All cells were stained with ThT (green fluorescence). **b** The ROS level of three samples tested by the microplate reader and stained with the DCFH-DA probe. The concentrations of two AIEgens and Aβ fibril were respectively 100 μg/mL and 10 μM. Data was presented as mean ± SD. **P* < 0.05, ****P* < 0.001, one-way ANOVA.

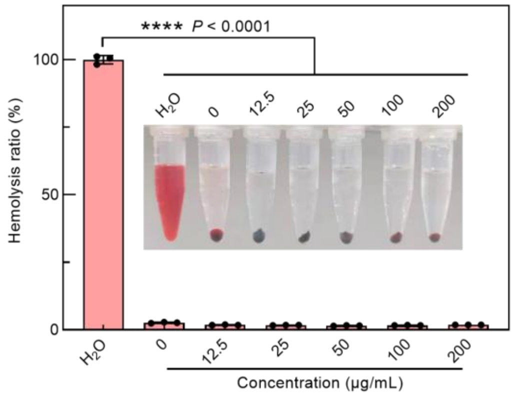

Supplementary Fig. 31 Hemolysis ratio and corresponding photograph (inset) of various groups. The erythrocyte treated with PBS was termed as 0. Data was presented as mean ± SD. *****P* < 0.0001, one-way ANOVA.

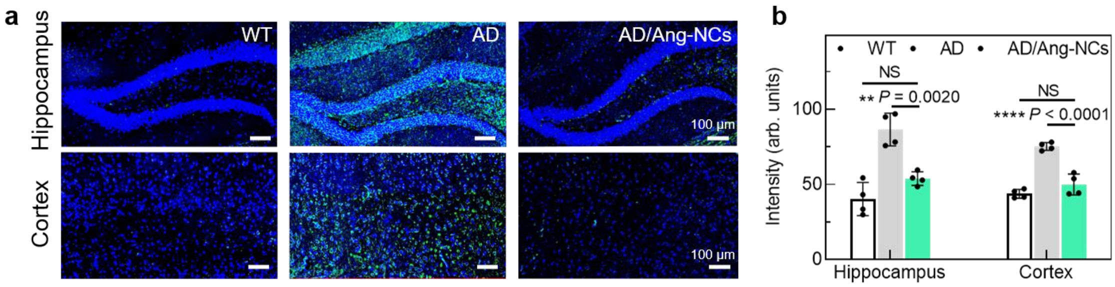

Supplementary Fig. 32 a Immunofluorescence images of 8-hydroxy-2'-deoxyguanosine (8-OHdG) in hippocampus and cortex regions of ex vivo brain slices from APP/PS1 mice (AD) and WT mice after treatment. **b** The corresponding fluorescence intensity quantification of 8-OHdG. Data was presented as mean ± SD. NS, no significance. ***P* < 0.01, *****P* < 0.0001, one-way ANOVA.

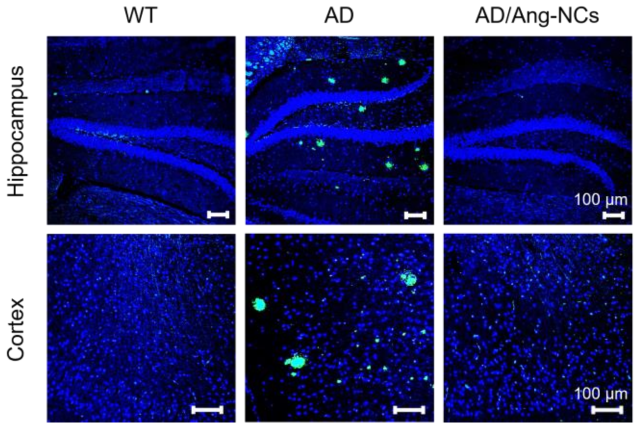

Supplementary Fig. 33 Microscopic observation of Aβ plaque (green spots) in hippocampus and cortex regions of ex vivo brain slices in WT mice, APP/PS1 (AD) mice, and AD mice with Ang-NCs. The green fluorescence was generated from the ThS dye.

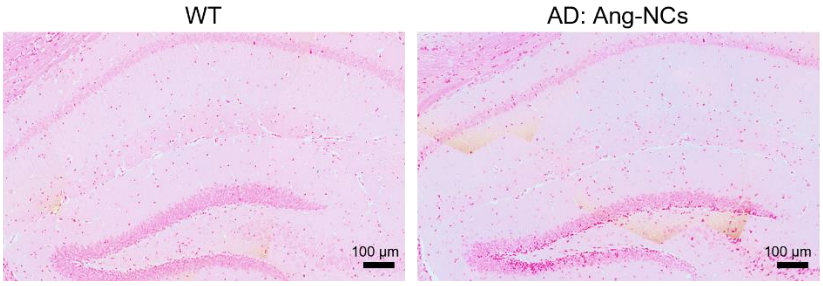

Supplementary Fig. 34 Microhemorrhage profile images of brain slices of AD mice treated with Ang-NCs and WT mice, both were stained with Prussian Blue Iron Stain Kit.

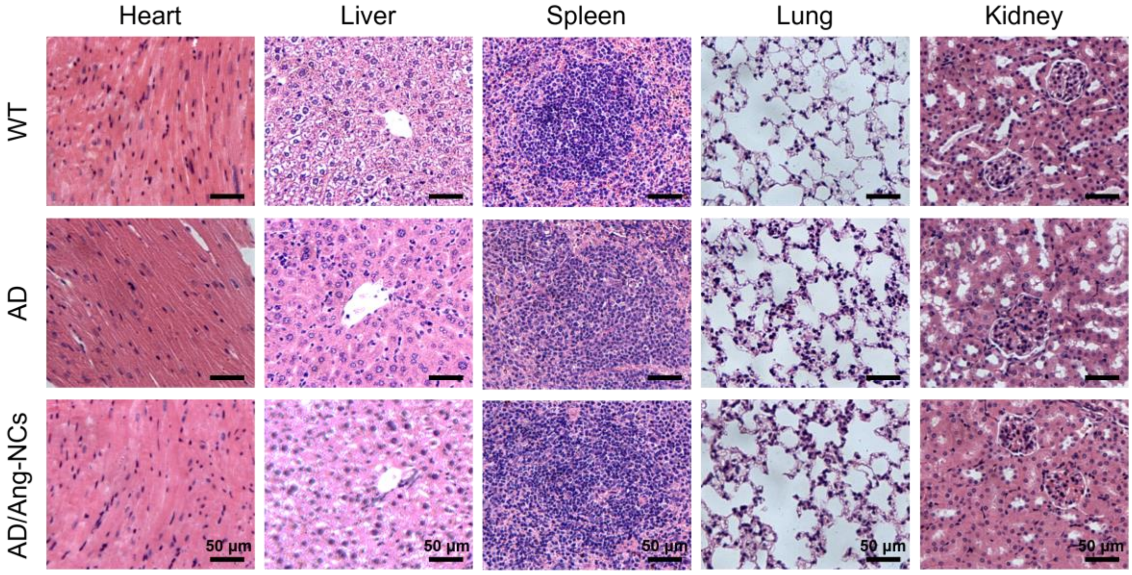

Supplementary Fig. 35 Hematoxylin and eosin (H&E) staining results for various organs of wild-type (WT) mice, APP/PS1 (AD) mice, and AD mice treated with Ang-NCs. All scale bars: 50 μm.

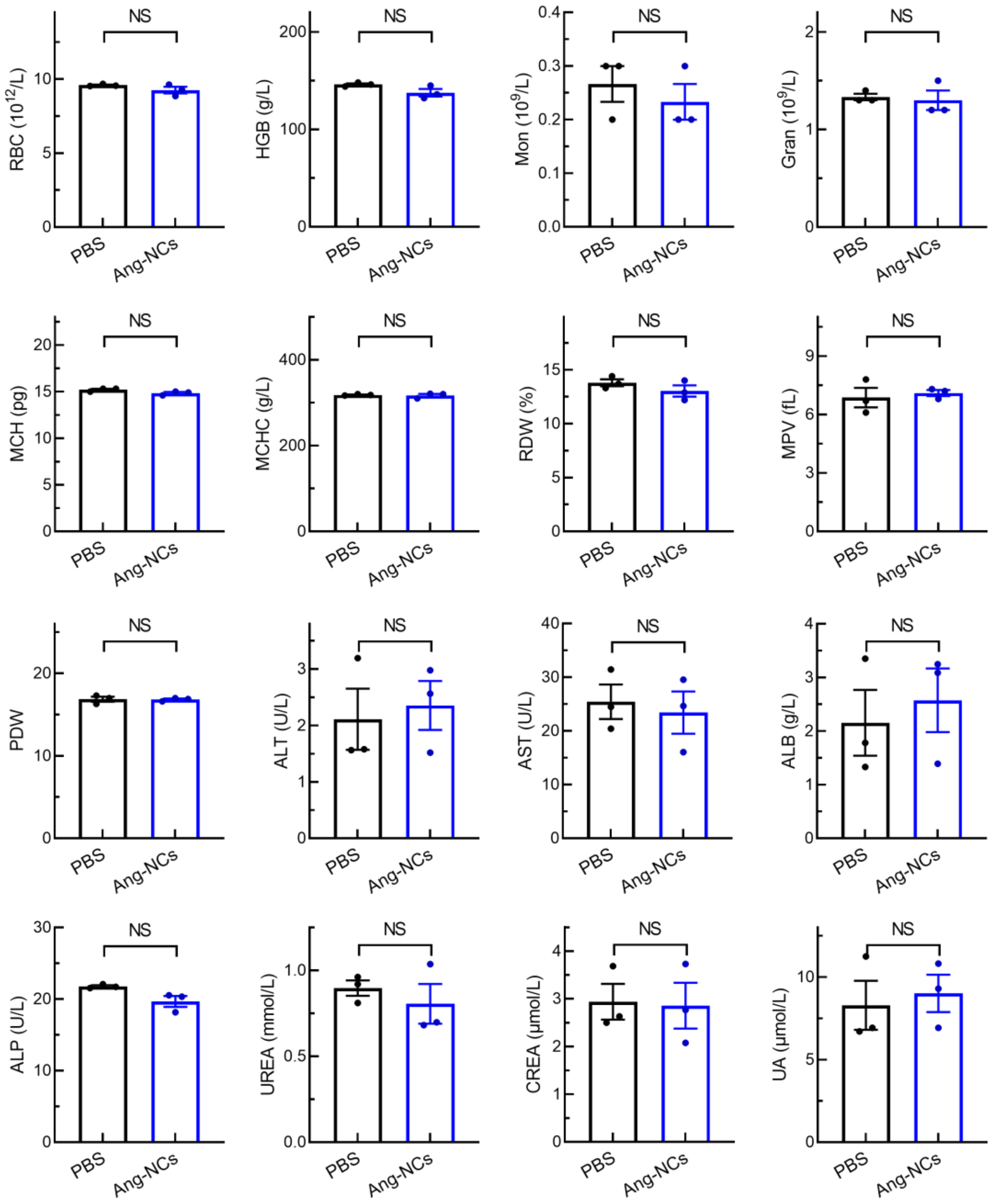

Supplementary Fig. 36 Blood routine examination and blood biochemistry. Blood was harvested from the healthy mice on the eighteenth day post i.v. injection of Ang-NCs (dosage: 10 mg/kg) and PBS six times (n = 3). Data were presented as mean ± SEM. NS, no significance, two-tailed Student's t-test.
